# Supplementary material for: The E3 ubiquitin-protein ligase Trim31 alleviates non-alcoholic fatty liver disease by targeting Rhbdf2 in mouse hepatocytes
Source: Nat Commun. 2022 Feb 25;13:1052. doi: 10.1038/s41467-022-28641-w (PMC8881609; doi:10.1038/s41467-022-28641-w)

The E3 ubiquitin-protein ligase Trim31 alleviates non-alcoholic fatty liver disease by targeting Rhbdf2 in mouse hepatocytes

Xu et al.

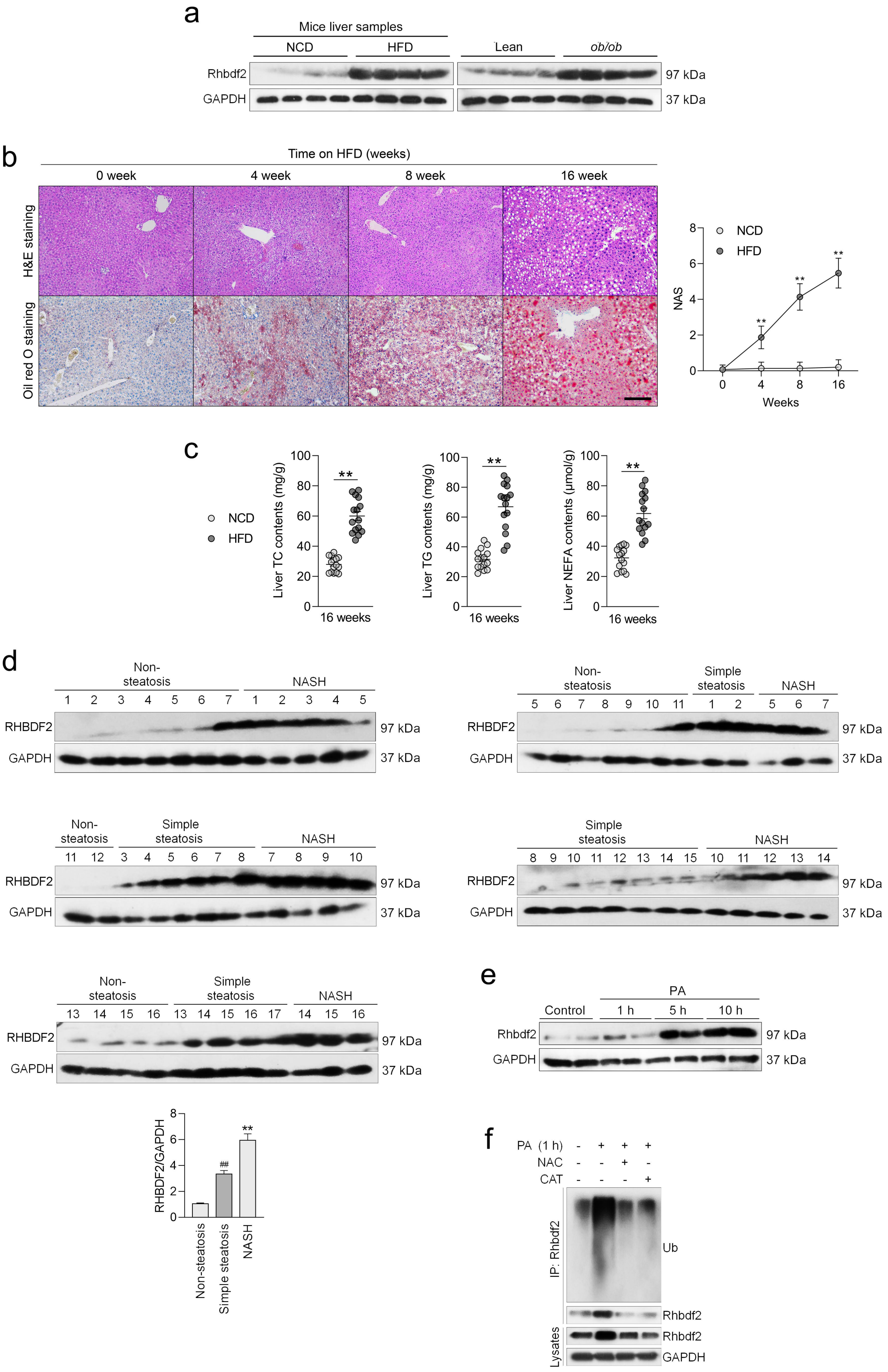
a
Mice liver samples
NCD
HFD
Lean
ob/ob
Rhbdf2
GAPDH
97 kDa
37 kDa
b
Time on HFD (weeks)
0 week
4 week
8 week
16 week
H&E staining
Oil red O staining
NCD
HFD
NAS
Weeks
c
Liver TC contents (mg/g)
Liver TG contents (mg/g)
Liver NEFA contents (μmol/g)
16 weeks
d
Non-steatosis
Simple steatosis
NASH
RHBDF2
GAPDH
RHBDF2/GAPDH
e
Control
PA
1 h
5 h
10 h
f
PA (1 h)
NAC
CAT
IP: Rhbdf2
Lysates
Ub

Supplementary Figure 1. Rhbdf2 is ubiquitinated, and Rhbdf2 protein expression is elevated in fatty liver. (**a**) Representative immunoblotting bands of Rhbdf2 expression in the liver samples isolated from C57BL/6N mice that were treated with a NCD or HFD for uninterrupted 16 weeks, or from *ob/ob* or lean mice (n=4 per experiment). (**b**) Representative images of H&E staining, Oil red O staining and NAS score evaluation for histological changes in the liver samples from HFD-fed mice (Magnification, 100×; n=5 images per group for each staining; n=15 for NAS score analysis) ($^{**}P < 0.01$ vs. NCD groups). (**c**) Records for the liver lipid contents including TG, TC and NEFA after 16 weeks-HFD fed mice (n=15 per experiment) ($^{**}P < 0.01$ vs. NCD groups). (**d**) Representative immunoblotting bands and relative expression levels of RHBDF2 in the liver samples of donors with non-steatosis (n=16 samples), simple steatosis (n=17 samples) or NASH (n=16 samples) phenotype ($^{**}P < 0.01$ vs. simple steatosis groups, $^{\#\#}P < 0.01$ vs. non-steatosis). (**e**) Representative immunoblotting bands of Rhbdf2 expression in cultured primary hepatocytes that were incubated with 400 μM palmitate (PA). The BSA was treated as controls (n=4 per experiment). (**f**) Representative immunoblotting results for ubiquitinated Rhbdf2 in cultured primary hepatocytes. Data are expressed as mean±SEM. The relevant experiments presented in this part were performed independently at least three times. Significance determined by Student's 2-tailed *t*-test analysis (b, c) and One-way analysis of variance (ANOVA) followed by Dunnett's multiple comparisons test (d).

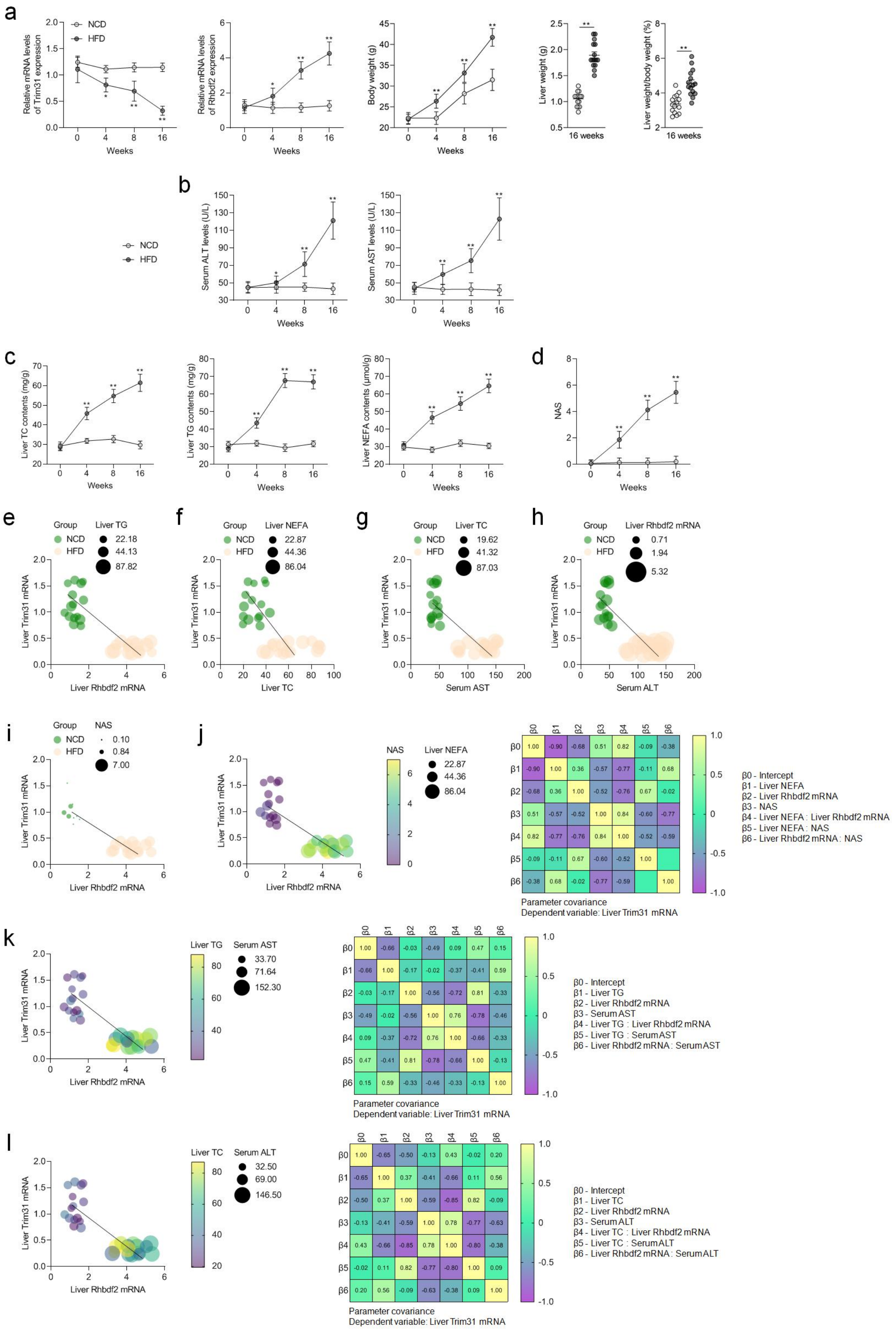

a
NCD
HFD
Relative mRNA levels of Trim31 expression
Relative mRNA levels of Rhbdf2 expression
Body weight (g)
Liver weight (g)
Liver weight/body weight (%)
Weeks
16 weeks
b
Serum ALT levels (U/L)
Serum AST levels (U/L)
c
Liver TC contents (mg/g)
Liver TG contents (mg/g)
Liver NEFA contents (μmol/g)
d
NAS
e
f
g
h
Group
Liver TG
Liver NEFA
Liver TC
Liver Rhbdf2 mRNA
Liver Trim31 mRNA
Serum AST
Serum ALT
i
j
k
l
Parameter covariance
Dependent variable: Liver Trim31 mRNA
β0 - Intercept
β1 - Liver NEFA
β2 - Liver Rhbdf2 mRNA
β3 - NAS
β4 - Liver NEFA : Liver Rhbdf2 mRNA
β5 - Liver NEFA : NAS
β6 - Liver Rhbdf2 mRNA : NAS
β1 - Liver TG
β3 - Serum AST
β4 - Liver TG : Liver Rhbdf2 mRNA
β5 - Liver TG : Serum AST
β6 - Liver Rhbdf2 mRNA : Serum AST
β1 - Liver TC
β3 - Serum ALT
β4 - Liver TC : Liver Rhbdf2 mRNA
β5 - Liver TC : Serum ALT
β6 - Liver Rhbdf2 mRNA : Serum ALT

Supplementary Figure 2. Correlation analysis of Trim31 expression levels-related parameters in HFD-fed mouse model. (**a-d**) Analysis results of relative Trim31 and Rhbdf2 mRNA expression levels, body weight, liver weight and liver weight/body weight ratio (a), serum ALT and AST contents (b), liver TG, TC and NEFA levels (c), and NAS score analysis in the liver samples isolated from C57BL/6N mice that were treated with a HFD over time (n=15 mice for each time point) ($^{*}P < 0.05$ and $^{**}P < 0.01$ vs. NCD groups). (**e-i**) Pearson correlation analyses indicating the correlations between mice liver Trim31 mRNA levels and Rhbdf2 mRNA levels (e), liver Trim31 mRNA levels and liver TC contents (f), liver Trim31 mRNA levels and serum AST levels (g), liver Trim31 mRNA levels and serum ALT levels (h), and liver Trim31 mRNA levels and NAS score (i), mRNA expression is normalized to GAPDH levels. $P < 0.001$ for all of these correlations (n=15 per indicator for each group; n=90 in total). (**j-l**) Multiple linear regression showing the correlations between alteration of mice liver samples Trim31 and Rhbdf2 mRNA levels and parameter indexes associating with liver NEFA/NAS score (j), liver TG/serum AST (k), and liver TC/serum ALT. Relative liver Trim31 mRNA expression is treated as dependent variable. $P < 0.001$ for all of these correlations (n=15 per indicator for each group; n=120 in total). Data are expressed as mean±SEM. The relevant experiments presented in this part were performed independently at least three times. Significance determined by Student's 2-tailed t-test analysis.

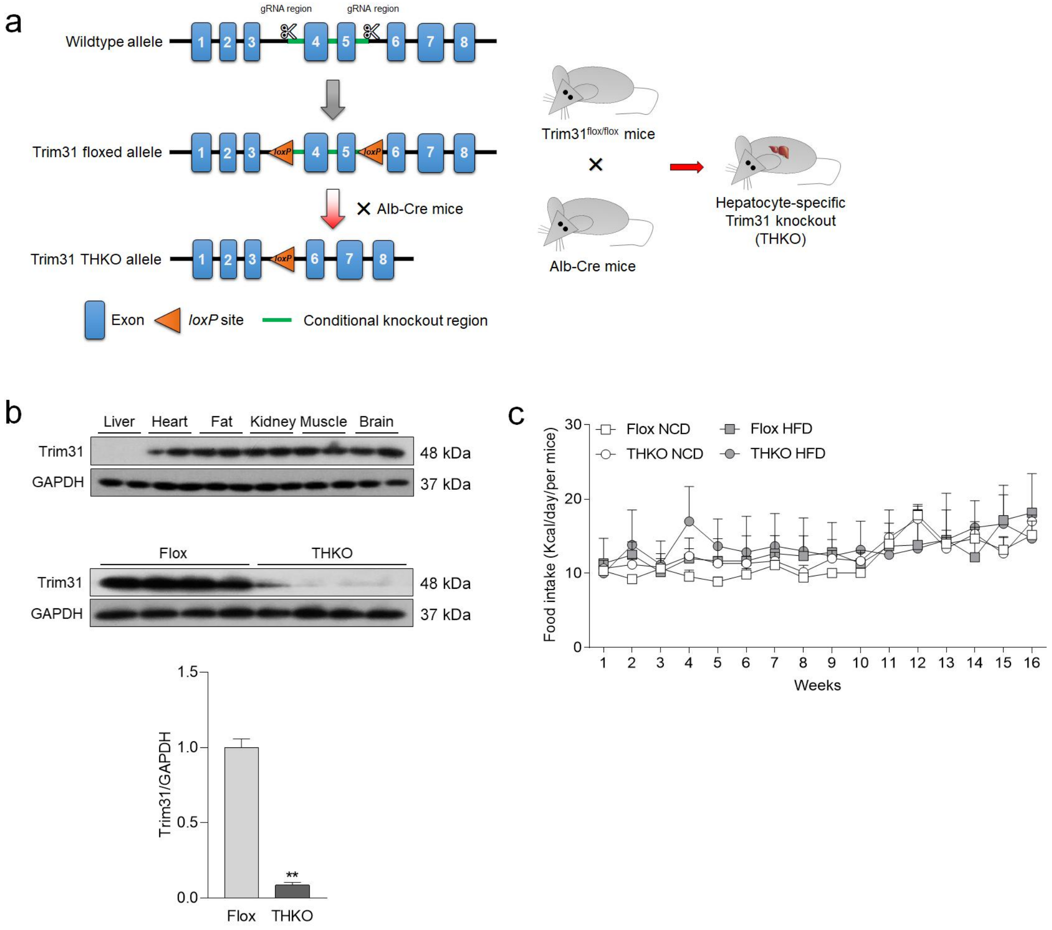
a
gRNA region
gRNA region
Wildtype allele
1 2 3 4 5 6 7 8
Trim31 floxed allele
loxP
× Alb-Cre mice
Trim31 THKO allele
Exon
loxP site
Conditional knockout region
Trim31flox/flox mice
×
Alb-Cre mice
Hepatocyte-specific
Trim31 knockout
(THKO)
b
Liver Heart Fat Kidney Muscle Brain
Trim31
GAPDH
48 kDa
37 kDa
Flox
THKO
Trim31/GAPDH
1.5
1.0
0.5
0.0
**
Flox THKO
c
Flox NCD
Flox HFD
THKO NCD
THKO HFD
Food intake (Kcal/day/per mice)
30
20
10
0
1 2 3 4 5 6 7 8 9 10 11 12 13 14 15 16
Weeks

Supplementary Figure 3. Liver-specific Trim31 deletion is unable to significantly alter energy consumption during HFD administration. (**a**) Schematic diagram showing the protocol for the generation of the hepatocyte-specific Trim31 deletion (THKO) mice. (**b**) Representative western blotting for the Trim31 expression in different organs (upper), and expression in the liver samples from Trim31-Flox and hepatocyte-specific Trim31 deletion (THKO) mice (lower) (n=4 per experiment) ($^{**}P < 0.01$ vs. Flox groups). (**c**) Records for the food intake (Kcal/day/per mice) of indicated mice in a 16-weeks HFD treatment (no significant difference). The GAPDH was used as loading control. Data are expressed as mean±SEM. The relevant experiments presented in this part were performed independently at least three times. Significance determined by Student's 2-tailed *t*-test analysis.

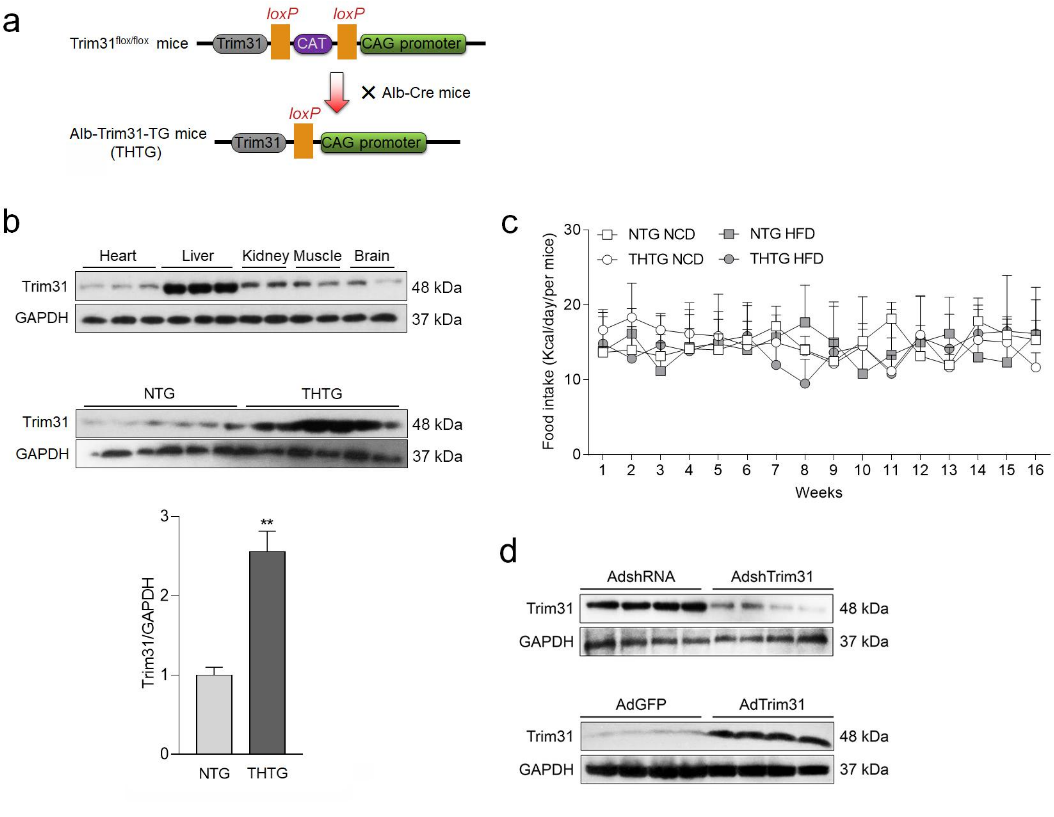
a
Trim31flox/flox mice
loxP
loxP
Trim31
CAT
CAG promoter
× Alb-Cre mice
Alb-Trim31-TG mice
(THTG)
loxP
Trim31
CAG promoter
b
Heart
Liver
Kidney
Muscle
Brain
Trim31
GAPDH
48 kDa
37 kDa
NTG
THTG
Trim31
GAPDH
48 kDa
37 kDa
Trim31/GAPDH
**
NTG
THTG
c
NTG NCD
THTG NCD
NTG HFD
THTG HFD
Food intake (Kcal/day/per mice)
Weeks
d
AdshRNA
AdshTrim31
Trim31
GAPDH
48 kDa
37 kDa
AdGFP
AdTrim31
Trim31
GAPDH
48 kDa
37 kDa

Supplementary Figure 4. Liver-specific Trim31 overexpression cannot change energy consumption during HFD treatment. (**a**) Schematic diagram showing the protocol for the generation of the hepatocyte-specific Trim31 transgenic (THTG) mice. (**b**) Representative western blotting for the Trim31 expression in different organs (upper) and in the liver samples from non-transgenic Trim31 (NTG) and THTG mice (lower) (*n*=6 per experiment) ($^{**}P < 0.01$ vs. NTG groups). (**c**) Records for the food intake (Kcal/day/per mice) of indicated mice in a 16-weeks HFD treatment (no significant difference). (**d**) Representative western blotting for the Trim31 expression efficiency in AdshTrim31 or AdTrim31-transfected primary hepatocytes (*n*=4 per experiment). The GAPDH was used as loading control. Data are expressed as mean±SEM. The relevant experiments presented in this part were performed independently at least three times. Significance determined by Student's 2-tailed *t*-test analysis.

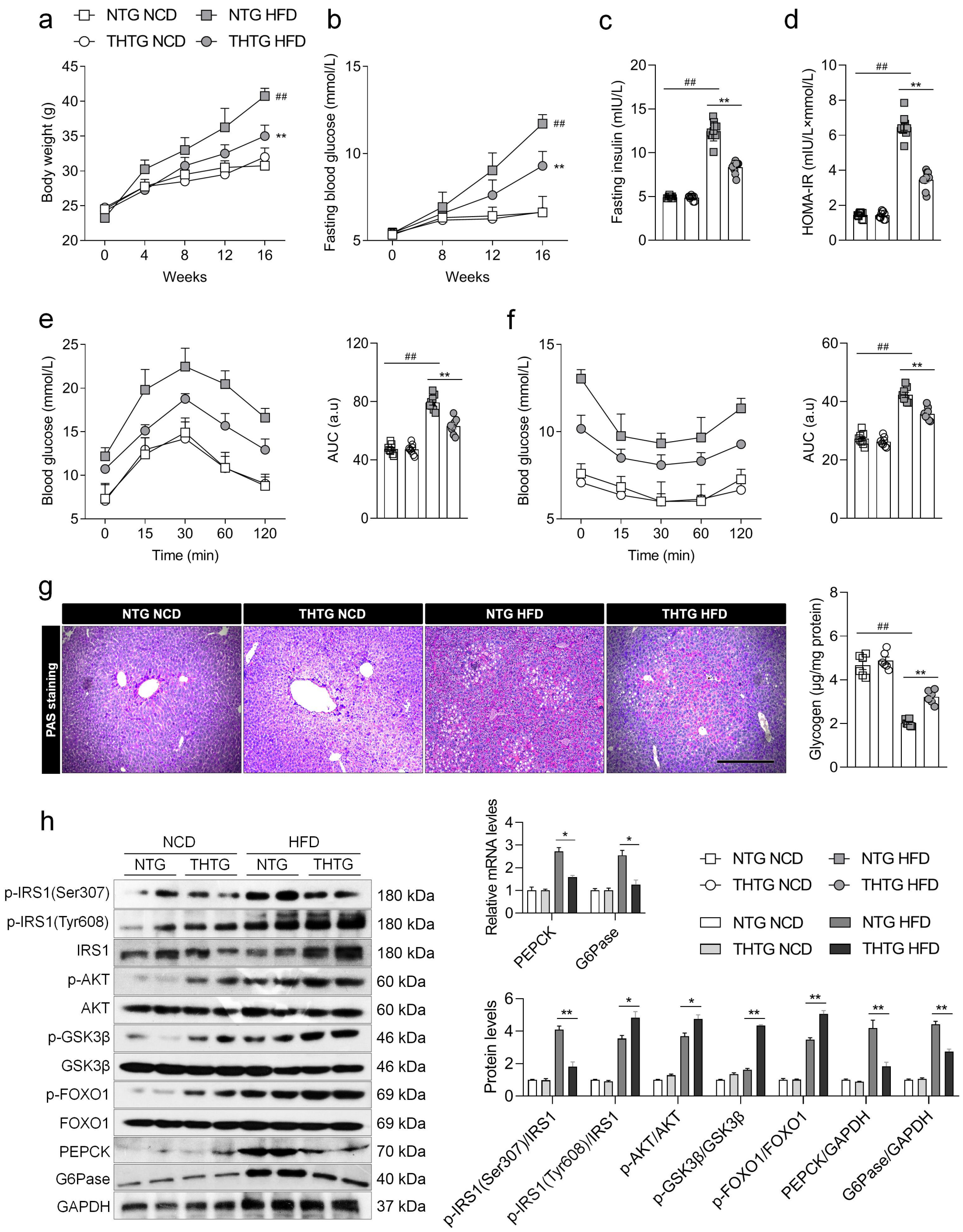

a
NTG NCD
NTG HFD
THTG NCD
THTG HFD
Body weight (g)
Weeks
##
**
b
Fasting blood glucose (mmol/L)
Weeks
##
**
c
Fasting insulin (mIU/L)
##
**
d
HOMA-IR (mIU/L×mmol/L)
##
**
e
Blood glucose (mmol/L)
Time (min)
AUC (a.u)
##
**
f
Blood glucose (mmol/L)
Time (min)
AUC (a.u)
##
**
g
NTG NCD
THTG NCD
NTG HFD
THTG HFD
PAS staining
Glycogen (μg/mg protein)
##
**
h
NCD
HFD
NTG
THTG
NTG
THTG
p-IRS1(Ser307) 180 kDa
p-IRS1(Tyr608) 180 kDa
IRS1 180 kDa
p-AKT 60 kDa
AKT 60 kDa
p-GSK3β 46 kDa
GSK3β 46 kDa
p-FOXO1 69 kDa
FOXO1 69 kDa
PEPCK 70 kDa
G6Pase 40 kDa
GAPDH 37 kDa
Relative mRNA levles
PEPCK
G6Pase
NTG NCD
THTG NCD
NTG HFD
THTG HFD
Protein levels
p-IRS1(Ser307)/IRS1
p-IRS1(Tyr608)/IRS1
p-AKT/AKT
p-GSK3β/GSK3β
p-FOXO1/FOXO1
PEPCK/GAPDH
G6Pase/GAPDH

Supplementary Figure 5. Hepatocyte-specific Trim31 overexpression ameliorates HFD-triggered pathological phenotypes of insulin resistance. (**a, b**) Body weight records (**a**) and fasting blood glucose levels (**b**) of non-transgenic Trim31 (NTG) and hepatocyte-specific Trim31 transgenic (THTG) mice during 16 weeks of NCD or HFD administration (n=15 mice) ($^{**}P < 0.01$ vs. NTG HFD groups, $^{\#\#}P < 0.01$ vs. NTG NCD groups). (**c, d**) Fasting insulin levels (**c**) and corresponding HOMA-IR index (**d**) of NTG and THTG mice at the last week of NCD or HFD feeding (n=10 mice) ($^{**}P < 0.01$ vs. NTG HFD groups, $^{\#\#}P < 0.01$ vs. NTG NCD groups). (**e, f**) Records for the glucose tolerance test (GTT) (**e**) and insulin tolerance test (ITT) (**f**) in the NTG and THTG mice at the last week of NCD or HFD ingestion; a.u, arbitrary unit (n=10 mice) ($^{**}P < 0.01$ vs. NTG HFD groups, $^{\#\#}P < 0.01$ vs. NTG NCD groups). (**g**) Representative images of Periodic Acid-Schiff stain (PAS)-stained pathological section for glycogen storage changes in the liver samples from the NTG and THTG mice (Magnification, 100×; n=6 images per group for each staining) ($^{**}P < 0.01$ vs. NTG HFD groups, $^{\#\#}P < 0.01$ vs. NTG NCD groups). (**h**) Representative immunoblotting bands for expression alterations of total amounts and phosphorylated forms of critical indicators involving in insulin signaling, including IRS1, AKT, GSK3β, FOXO1, PEPCK and G6Pase, in the liver of NTG and THTG mice after HFD feeding for 16 weeks (n=4 per experiment) ($^{*}P < 0.05$ and $^{**}P < 0.01$ vs. NTG HFD groups). The GAPDH was used as loading control. Data are expressed as mean±SEM. The relevant experiments presented in this part were performed independently at least three times. Significance determined by two-way analysis of variance with general linear model procedures using a univariate approach.

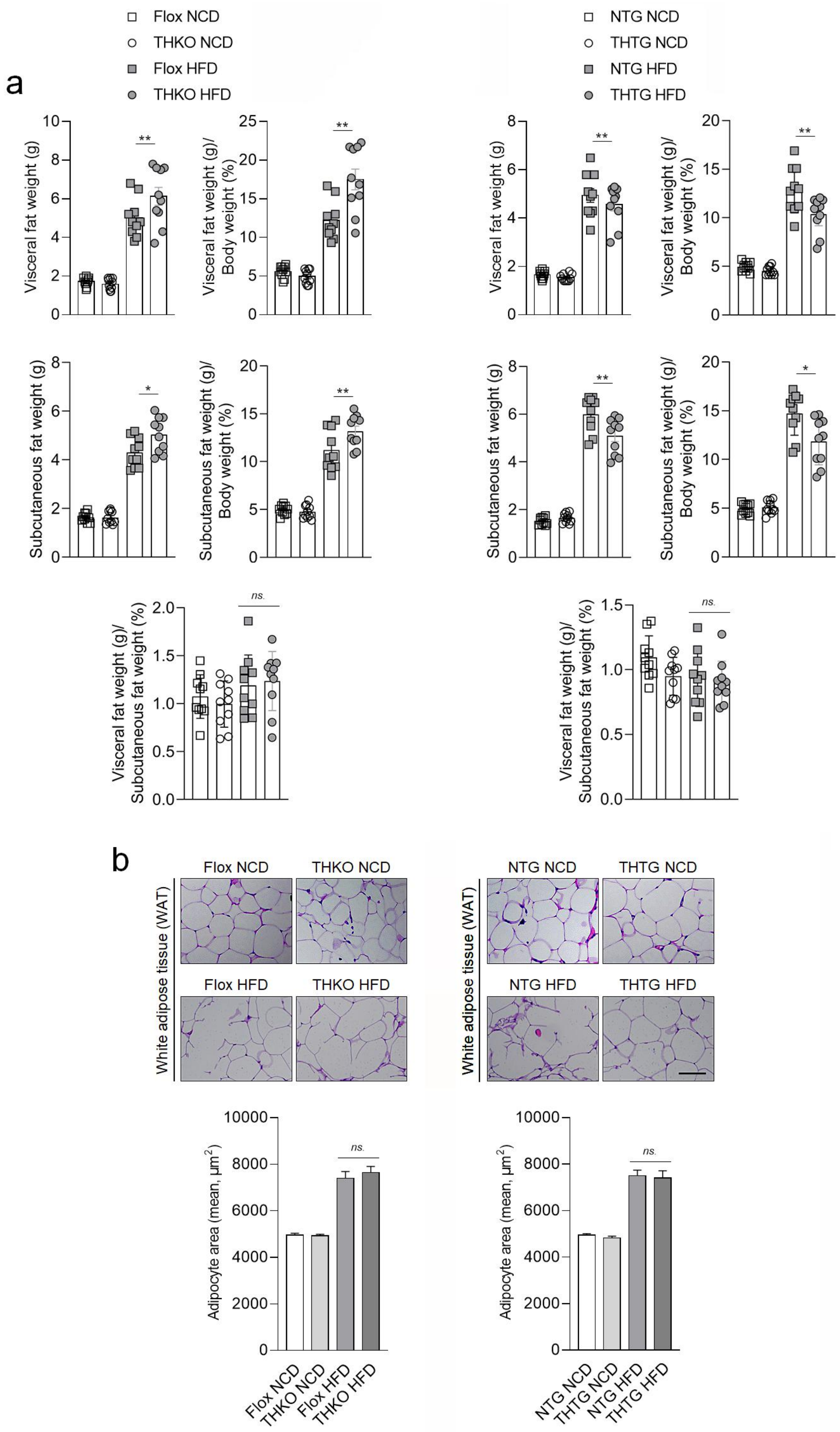

a
Flox NCD
THKO NCD
Flox HFD
THKO HFD
NTG NCD
THTG NCD
NTG HFD
THTG HFD
Visceral fat weight (g)
Visceral fat weight (g)/ Body weight (%)
Subcutaneous fat weight (g)
Subcutaneous fat weight (g)/ Body weight (%)
Visceral fat weight (g)/ Subcutaneous fat weight (%)
ns.
b
White adipose tissue (WAT)
Adipocyte area (mean, μm²)

Supplementary Figure 6. Trim31 deficiency promotes weight gain of adipose tissue to facilitate HFD-triggered mice obesity. (**a**) Visceral fat weights, visceral fat weight/body weight ratio, subcutaneous fat weights, subcutaneous fat/body weight ratio and visceral fat weight/subcutaneous fat weight ratio in the hepatocyte-specific Trim31 deletion (THKO) mice, hepatocyte-specific Trim31 transgenic (THTG) mice, and their corresponding controls (Flox- and NTG-mice) after a 16-weeks of HFD or NCD treatment (n=10 mice per group) ($^{*}P < 0.05$ and $^{**}P < 0.01$ vs. NTG HFD groups or Flox HFD groups, *ns.*, no significant difference). (**b**) The representative H&E staining pictures and the analyzed adipocytes area (μm^2) on the white adipose tissue (WAT) sections of mice in the indicated groups (Magnification, 200×; n=10 images per group for each staining) (*ns.*, no significant difference). Data are expressed as mean±SEM. The relevant experiments presented in this part were performed independently at least three times. Significance determined by one-way ANOVA followed by Dunnett's multiple comparisons test.

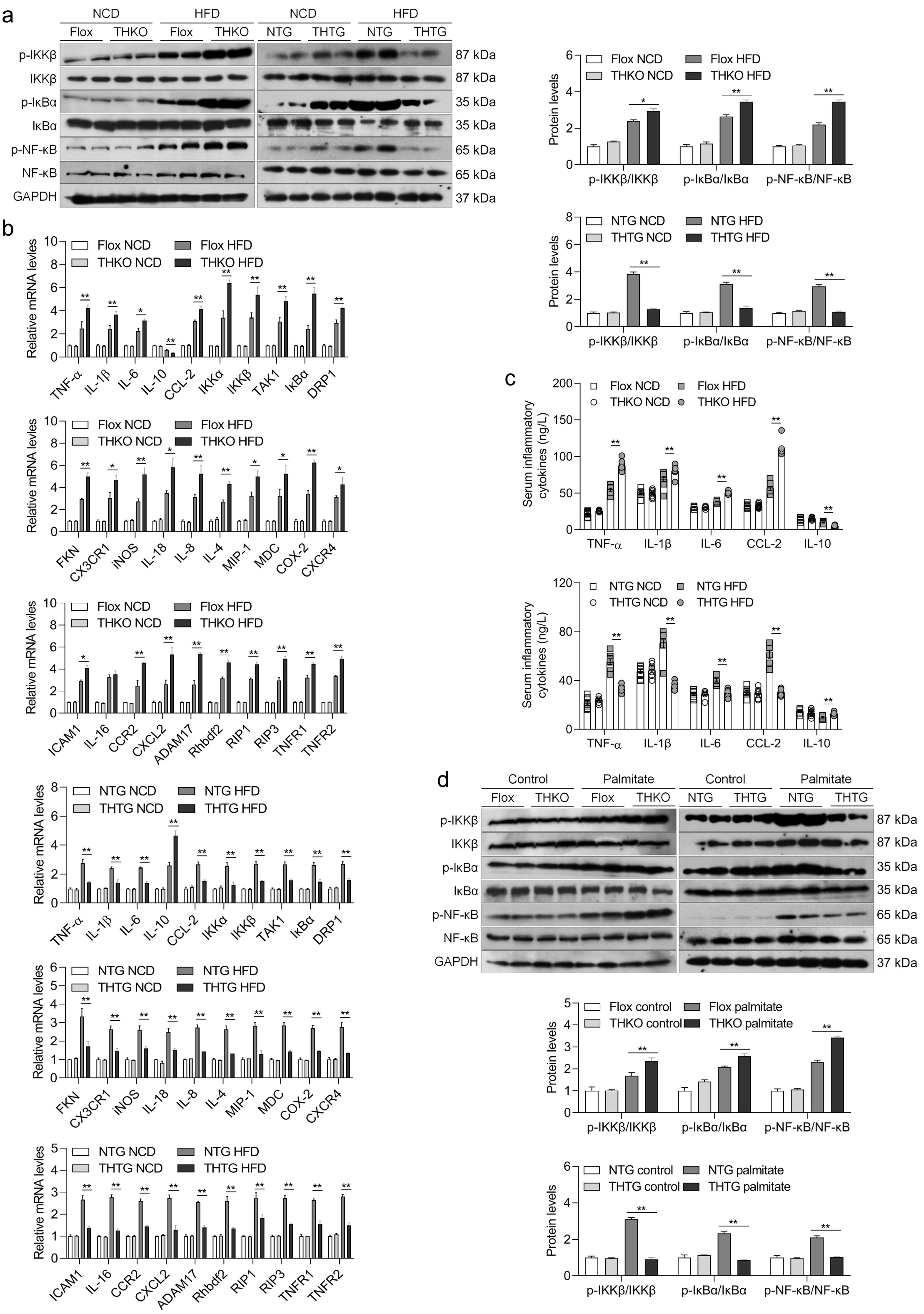

a
NCD
HFD
NCD
HFD
Flox
THKO
Flox
THKO
NTG
THTG
NTG
THTG
p-IKKβ
IKKβ
p-IκBα
IκBα
p-NF-κB
NF-κB
GAPDH
87 kDa
87 kDa
35 kDa
35 kDa
65 kDa
65 kDa
37 kDa
Flox NCD
Flox HFD
THKO NCD
THKO HFD
Protein levels
p-IKKβ/IKKβ
p-IκBα/IκBα
p-NF-κB/NF-κB
NTG NCD
NTG HFD
THTG NCD
THTG HFD
b
Relative mRNA levles
TNF-α
IL-1β
IL-6
IL-10
CCL-2
IKKα
IKKβ
TAK1
IκBα
DRP1
FKN
CX3CR1
iNOS
IL-18
IL-8
IL-4
MIP-1
MDC
COX-2
CXCR4
ICAM1
IL-16
CCR2
CXCL2
ADAM17
Rhbdf2
RIP1
RIP3
TNFR1
TNFR2
c
Serum inflammatory cytokines (ng/L)
Flox NCD
Flox HFD
THKO NCD
THKO HFD
NTG NCD
NTG HFD
THTG NCD
THTG HFD
TNF-α
IL-1β
IL-6
CCL-2
IL-10
d
Control
Palmitate
Control
Palmitate
Flox
THKO
Flox
THKO
NTG
THTG
NTG
THTG
Flox control
Flox palmitate
THKO control
THKO palmitate
NTG control
NTG palmitate
THTG control
THTG palmitate
Protein levels
p-IKKβ/IKKβ
p-IκBα/IκBα
p-NF-κB/NF-κB

Supplementary Figure 7. Functional loss of hepatic Trim31 aggravates HFD-induced liver inflammation. (**a**) Representative immunoblotting bands for expression alterations of total amounts or phosphorylated forms of critical indicators associating with inflammation signaling including IKKβ, p-IKKβ, IκBα, p-IκBα, NF-κB and p-NF-κB in the liver samples from THKO-, THTG-, NTG-, Flox mice after 16-weeks HFD or NCD feeding (n=4 per experiment). (**b**) qPCR analysis of the relative mRNA expression of genes associated with inflammation signaling in the liver samples from THKO-, THTG-, NTG-, Flox mice after 16-weeks HFD or NCD feeding (n=10 liver samples per group) ($^{*}P < 0.05$ and $^{**}P < 0.01$ vs. Flox HFD groups or NTG HFD groups). (**c**) Representative pro-inflammatory cytokines and chemokines levels including TNF-α, IL-1β, IL-6, CCL-2, and IL-10 in serum from THKO-, THTG-, NTG-, Flox mice after 16-weeks HFD or NCD feeding (n=10 mice per group) ($^{*}P < 0.05$ and $^{**}P < 0.01$ vs. Flox HFD groups or NTG HFD groups). (**d**) Representative immunoblotting bands for expression changes of total amounts or phosphorylated forms of critical indicators associating with inflammation signaling including IKKβ, p-IKKβ, IκBα, p-IκBα, NF-κB and p-NF-κB in primary hepatocytes isolated from the Flox-, THKO- NTG- and THTG-mice that were incubated with 400 μM palmitate (PA) for 10 hours (n=4 per experiment) ($^{**}P < 0.01$ vs. Flox palmitate groups or NTG palmitate groups). The GAPDH was used as loading control. Data are expressed as mean±SEM. The relevant experiments presented in this part were performed independently at least three times. Significance determined by one-way ANOVA followed by Dunnett's multiple comparisons test.

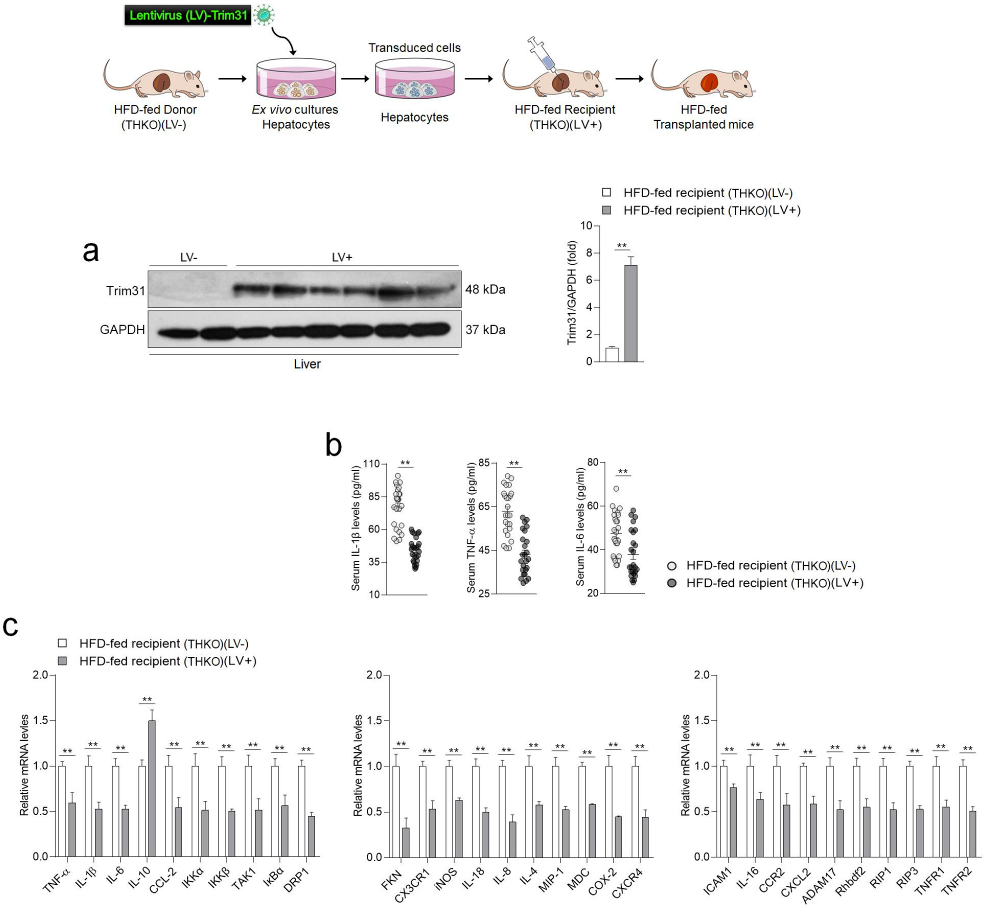
Lentivirus (LV)-Trim31
Transduced cells
HFD-fed Donor (THKO)(LV-)
Ex vivo cultures Hepatocytes
Hepatocytes
HFD-fed Recipient (THKO)(LV+)
HFD-fed Transplanted mice
a
LV-
LV+
Trim31
48 kDa
GAPDH
37 kDa
Liver
HFD-fed recipient (THKO)(LV-)
HFD-fed recipient (THKO)(LV+)
Trim31/GAPDH (fold)
**
b
Serum IL-1β levels (pg/ml)
Serum TNF-α levels (pg/ml)
Serum IL-6 levels (pg/ml)
HFD-fed recipient (THKO)(LV-)
HFD-fed recipient (THKO)(LV+)
c
HFD-fed recipient (THKO)(LV-)
HFD-fed recipient (THKO)(LV+)
Relative mRNA levles
TNF-α
IL-1β
IL-6
IL-10
CCL-2
IKKα
IKKβ
TAK1
IκBα
DRP1
FKN
CX3CR1
iNOS
IL-18
IL-8
IL-4
MIP-1
MDC
COX-2
CXCR4
ICAM1
IL-16
CCR2
CXCL2
ADAM17
Rhbdf2
RIP1
RIP3
TNFR1
TNFR2

Supplementary Figure 8. Allogeneic hepatocyte transplantation using lentivirus-mediated Trim31 expression mitigates HFD-triggered liver inflammation. Preconditioned liver-specific Trim31 deletion (THKO) mice with an 8-weeks HFD treatment as donor were transduced (THKO)(LV-). The hepatocytes isolated from (THKO)(LV-) group mice were transduced with lentivirus-loaded full-length Trim31 sequence. The corresponding blank vector were transduced as controls. Then, the additional HFD-fed THKO mice as recipient were injected with transduced hepatocytes via portal vein. The HFD-fed transplanted (THKO)(LV+) mice were harvested for the further experimental detection. (**a**) Increased Trim31 expression in liver tissue after transplantation were detected by western blotting (*n*=6 per experiment) ($^{**}P$ < 0.01 vs. HFD-fed recipient (THKO)(LV-) groups). (**b**) Representative pro-inflammatory cytokines including TNF-α, IL-1β and IL-6 in serum from HFD-fed transplanted (THKO)(LV+) and HFD-fed transplanted (THKO)(LV-) mice after 16-weeks HFD feeding (*n*=25 mice per group) ($^{**}P$ < 0.01 vs. HFD-fed recipient (THKO)(LV-) groups). (**c**) qPCR analysis of the relative mRNA expression of genes associated with inflammation signaling in the liver samples from HFD-fed transplanted (THKO)(LV+) and HFD-fed transplanted (THKO)(LV-) mice after 16-weeks HFD feeding (*n*=10 liver samples per group) ($^{**}P$ < 0.01 vs. HFD-fed recipient (THKO)(LV-) groups). The GAPDH was used as loading control. Data are expressed as mean±SEM. The relevant experiments presented in this part were performed independently at least three times. Significance determined by Student's 2-tailed *t*-test analysis.

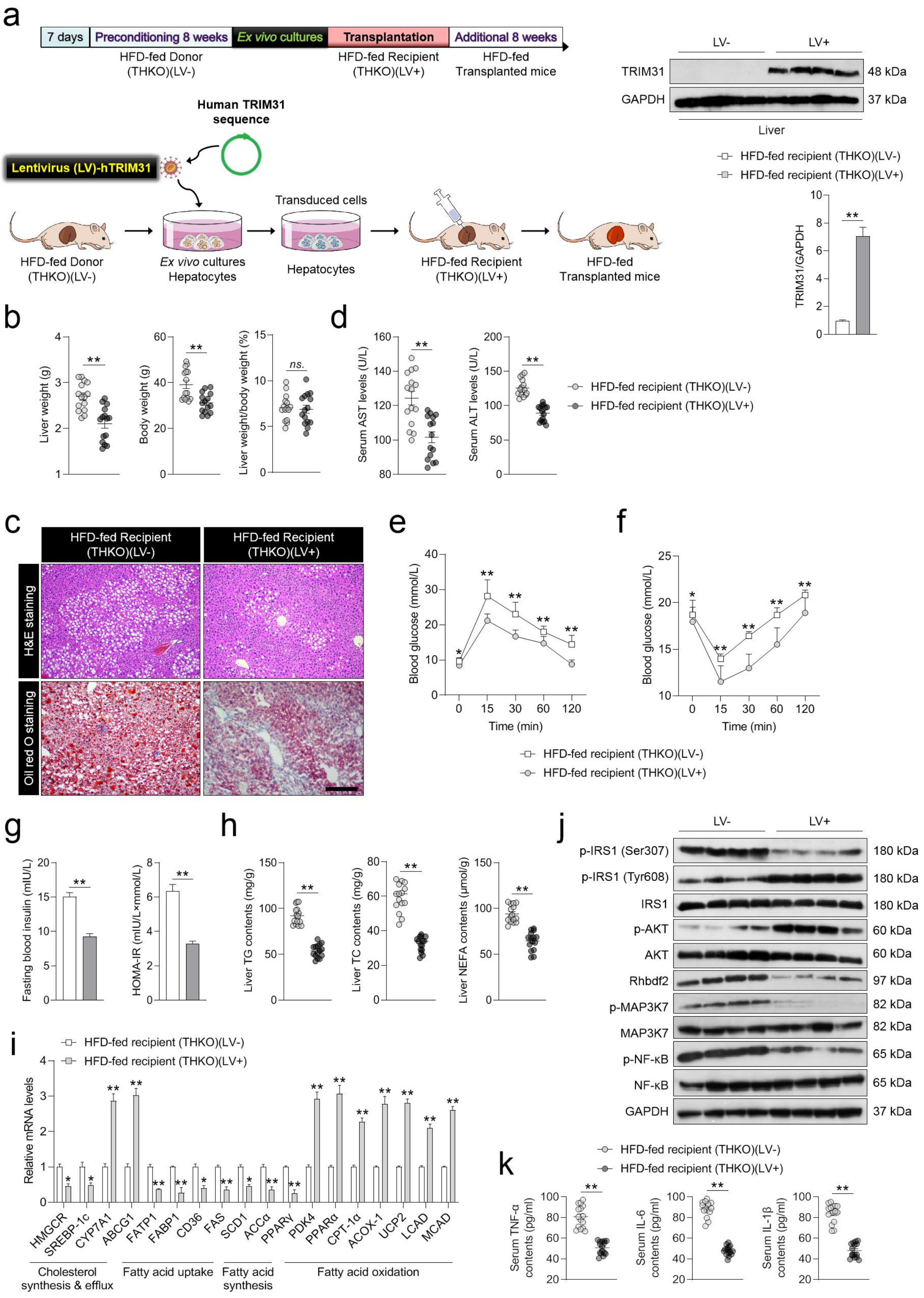

a
7 days
Preconditioning 8 weeks
Ex vivo cultures
Transplantation
Additional 8 weeks
HFD-fed Donor (THKO)(LV-)
HFD-fed Recipient (THKO)(LV+)
HFD-fed Transplanted mice
Human TRIM31 sequence
Lentivirus (LV)-hTRIM31
Transduced cells
HFD-fed Donor (THKO)(LV-)
Ex vivo cultures Hepatocytes
Hepatocytes
HFD-fed Recipient (THKO)(LV+)
HFD-fed Transplanted mice
LV-
LV+
TRIM31
48 kDa
GAPDH
37 kDa
Liver
HFD-fed recipient (THKO)(LV-)
HFD-fed recipient (THKO)(LV+)
TRIM31/GAPDH
b
Liver weight (g)
Body weight (g)
Liver weight/body weight (%)
ns.
d
Serum AST levels (U/L)
Serum ALT levels (U/L)
HFD-fed recipient (THKO)(LV-)
HFD-fed recipient (THKO)(LV+)
c
HFD-fed Recipient (THKO)(LV-)
HFD-fed Recipient (THKO)(LV+)
H&E staining
Oil red O staining
e
Blood glucose (mmol/L)
Time (min)
f
Blood glucose (mmol/L)
Time (min)
HFD-fed recipient (THKO)(LV-)
HFD-fed recipient (THKO)(LV+)
g
Fasting blood insulin (mIU/L)
HOMA-IR (mIU/L×mmol/L)
h
Liver TG contents (mg/g)
Liver TC contents (mg/g)
Liver NEFA contents (μmol/g)
j
LV-
LV+
p-IRS1 (Ser307) 180 kDa
p-IRS1 (Tyr608) 180 kDa
IRS1 180 kDa
p-AKT 60 kDa
AKT 60 kDa
Rhbdf2 97 kDa
p-MAP3K7 82 kDa
MAP3K7 82 kDa
p-NF-κB 65 kDa
NF-κB 65 kDa
GAPDH 37 kDa
i
HFD-fed recipient (THKO)(LV-)
HFD-fed recipient (THKO)(LV+)
Relative mRNA levels
HMGCR
SREBP-1c
CYP7A1
ABCG1
FATP1
FABP1
CD36
FAS
SCD1
ACCα
PPARγ
PDK4
PPARα
CPT-1α
ACOX-1
UCP2
LCAD
MCAD
Cholesterol synthesis & efflux
Fatty acid uptake
Fatty acid synthesis
Fatty acid oxidation
k
HFD-fed recipient (THKO)(LV-)
HFD-fed recipient (THKO)(LV+)
Serum TNF-α contents (pg/ml)
Serum IL-6 contents (pg/ml)
Serum IL-1β contents (pg/ml)

Supplementary Figure 9. Allogeneic hepatocyte transplantation using lentivirus-mediated human TRIM31 alleviates HFD-triggered hepatic steatosis, insulin resistance and inflammation. Preconditioned liver-specific Trim31 deletion (THKO) mice with an 8-weeks HFD treatment as donor were transduced (THKO)(LV-). The hepatocytes isolated from (THKO)(LV-) group mice were transduced with lentivirus-loaded human full-length TRIM31 sequence. The corresponding blank vector were transduced as controls. Then, the additional HFD-fed THKO mice as recipient were injected with transduced hepatocytes via portal vein. The HFD-fed transplanted (THKO)(LV+) mice were harvested for the further experimental detection. (**a**) The strategy diagram for *ex vivo* gene therapy used in the current study ($^{**}P < 0.01$ vs. HFD-fed recipient (THKO)(LV-) groups). (**b**) Records for the liver weight, body weight and the ratio of liver weight/body weight (%) of the HFD-fed recipient (THKO)(LV-) and (THKO)(LV+) mice at the last week of HFD treatment (*n*=15 mice per group) ($^{**}P < 0.01$ vs. HFD-fed recipient (THKO)(LV-) groups, *ns.*, no significant difference). (**c**) Representative pictures for Oil red O staining and H&E staining-indicated liver histopathologic changes of the HFD-fed recipient (THKO)(LV-) and (THKO)(LV+) mice after *ex vivo* experiment (Magnification, 100×; *n*=10 images per group for each staining). (**d**) Liver function markers ALT and AST levels were detected in transduced recipient mice (*n*=15 mice per group) ($^{**}P < 0.01$ vs. HFD-fed recipient (THKO)(LV-) groups). (**e-g**) Records for the glucose tolerance test (GTT) (e) and insulin tolerance test (ITT) (f), and the corresponding fasting insulin levels and HOMA-IR index (g) in the HFD-fed recipient (THKO)(LV-) and (THKO)(LV+) mice after transplantation (*n*=15 mice per group) ($^{**}P < 0.01$ vs. HFD-fed recipient (THKO)(LV-) groups). (**h**) Liver lipid contents including TG, TC and NEFA of the transduced mice (*n*=15 mice per group) ($^{**}P < 0.01$ vs. HFD-fed recipient (THKO)(LV-) groups). (**i**) qPCR analysis of the relative mRNA expression of genes associated with fatty acid uptake, synthesis, and β-oxidation in the HFD-fed recipient (THKO)(LV-) and (THKO)(LV+) mice after transplantation (*n*=10 liver samples per group) ($^{**}P < 0.01$ vs. HFD-fed recipient (THKO)(LV-) groups). (**j**) Representative immunoblotting bands for expression alterations of total amounts and phosphorylated forms of critical indicators involving in insulin signaling, including IRS1, p-IRS1(Ser307), p-IRS1(Tyr608), AKT, and p-AKT, and Rhbdf2-MAP3K7 axis and p-NF-κB pathway in the liver of HFD-fed recipient (THKO)(LV-) and

(THKO)(LV+) mice. (**k**) Pro-inflammatory cytokines levels (TNF-α, IL-6 and IL-1β) in serum in the liver of HFD-fed recipient (THKO)(LV-) and (THKO)(LV+) mice ($^{**}P$ < 0.01 vs. HFD-fed recipient (THKO)(LV-) groups). The GAPDH was used as loading control. Data are expressed as mean±SEM. The relevant experiments presented in this part were performed independently at least three times. Significance determined by Student's 2-tailed *t*-test analysis.

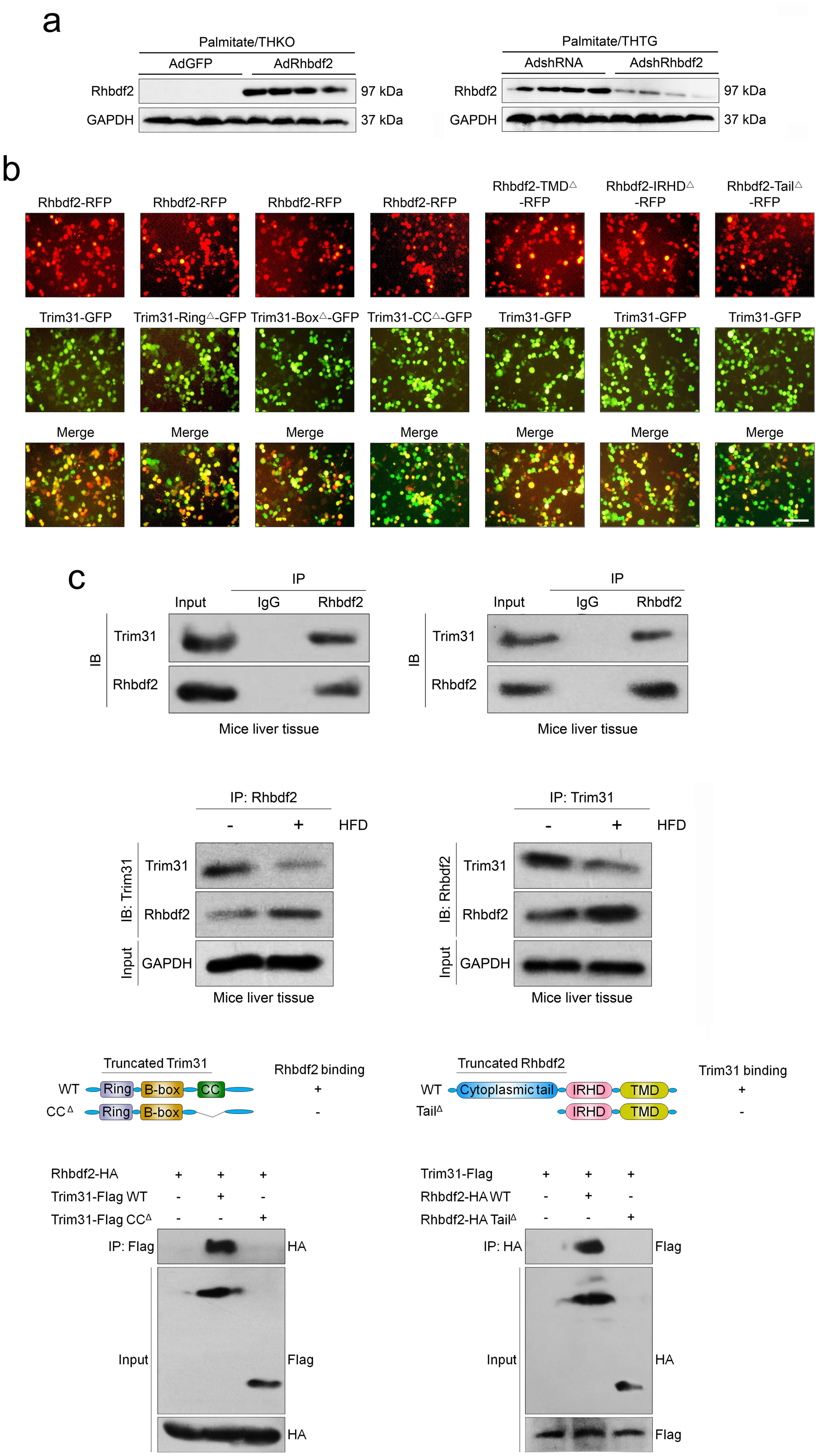
a
Palmitate/THKO
AdGFP
AdRhbdf2
Rhbdf2
97 kDa
GAPDH
37 kDa
Palmitate/THTG
AdshRNA
AdshRhbdf2
Rhbdf2
97 kDa
GAPDH
37 kDa
b
Rhbdf2-RFP
Rhbdf2-RFP
Rhbdf2-RFP
Rhbdf2-RFP
Rhbdf2-TMD△-RFP
Rhbdf2-IRHD△-RFP
Rhbdf2-Tail△-RFP
Trim31-GFP
Trim31-Ring△-GFP
Trim31-Box△-GFP
Trim31-CC△-GFP
Trim31-GFP
Trim31-GFP
Trim31-GFP
Merge
Merge
Merge
Merge
Merge
Merge
Merge
c
IP
Input
IgG
Rhbdf2
IB
Trim31
Rhbdf2
Mice liver tissue
IP
Input
IgG
Rhbdf2
IB
Trim31
Rhbdf2
Mice liver tissue
IP: Rhbdf2
- + HFD
IB: Trim31
Trim31
Rhbdf2
Input
GAPDH
Mice liver tissue
IP: Trim31
- + HFD
IB: Rhbdf2
Trim31
Rhbdf2
Input
GAPDH
Mice liver tissue
Truncated Trim31
Rhbdf2 binding
WT Ring B-box CC +
CC△ Ring B-box -
Truncated Rhbdf2
Trim31 binding
WT Cytoplasmic tail IRHD TMD +
Tail△ IRHD TMD -
Rhbdf2-HA + + +
Trim31-Flag WT - + -
Trim31-Flag CC△ - - +
IP: Flag
HA
Input
Flag
HA
Trim31-Flag + + +
Rhbdf2-HA WT - + -
Rhbdf2-HA Tail△ - - +
IP: HA
Flag
Input
HA
Flag

Supplementary Figure 10. The transfection efficiency of adenovirus-induced hepatocytes and protein interaction between mice Rhbdf2 and Trim31. (**a**) The primary hepatocytes isolated from the THKO or THTG mice were transfected with AdRhbdf2 or AdshRhbdf2. The upper western blotting bands shows the expression efficiency of Rhbdf2. The lower western blotting bands shows the inhibitory efficiency of Rhbdf2 (n=4 per experiment). (**b**) The L02 cells co-transfected with expression vectors for different sizes of the truncated target proteins (Magnification, 200×; n=5 images per group for each staining). (**c**) The binding domains of mice Rhbdf2 and Trim31 were detected using full-length and truncated Rhbdf2 or Trim31 expression vectors based on IP assays. Anti-Flag or anti-HA antibodies were used to confirm the binding sites of Rhbdf2 and Trim31, respectively.

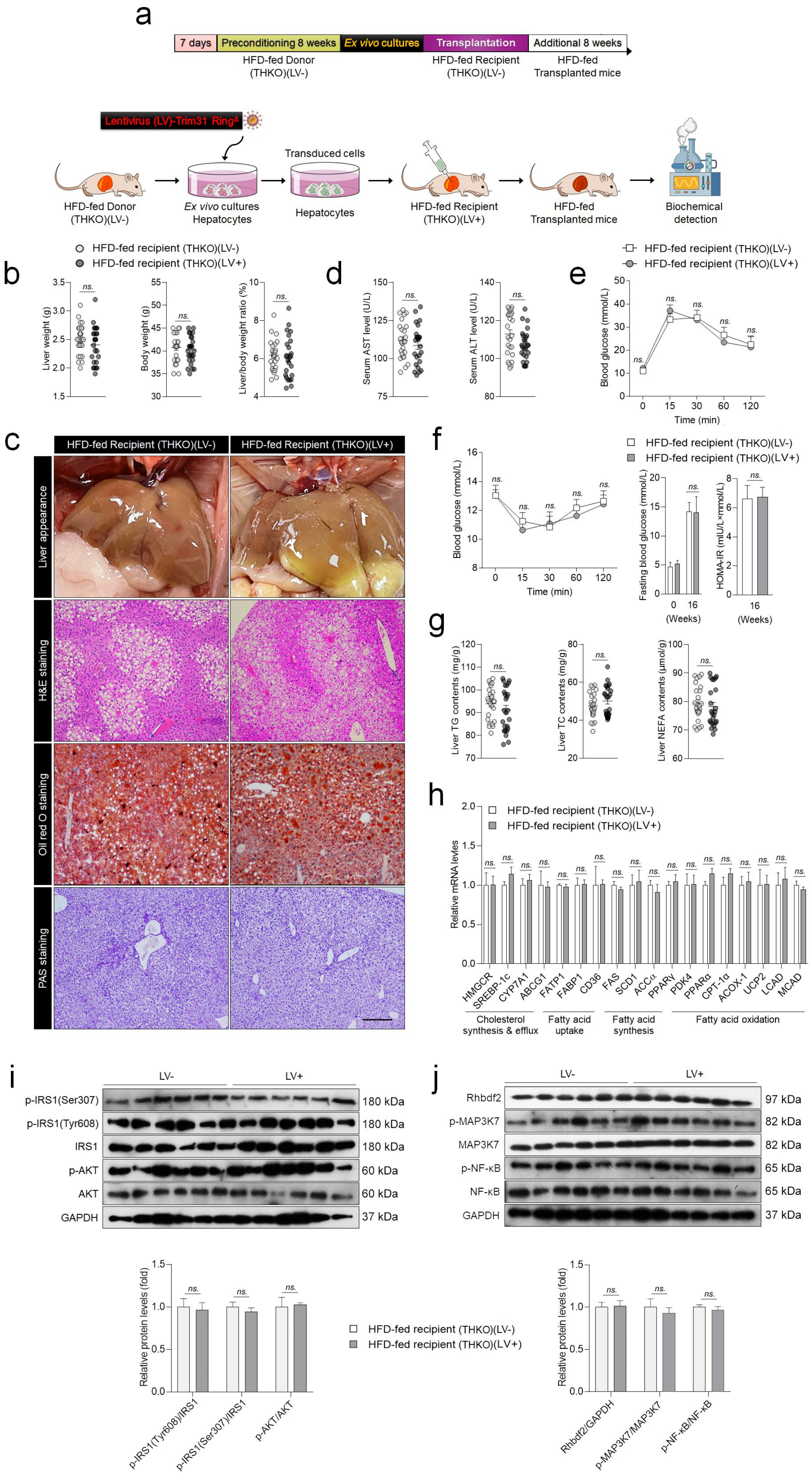
a
7 days
Preconditioning 8 weeks
Ex vivo cultures
Transplantation
Additional 8 weeks
HFD-fed Donor (THKO)(LV-)
HFD-fed Recipient (THKO)(LV-)
HFD-fed Transplanted mice
Lentivirus (LV)-Trim31 Ring^Δ
Transduced cells
HFD-fed Donor (THKO)(LV-)
Ex vivo cultures Hepatocytes
Hepatocytes
HFD-fed Recipient (THKO)(LV+)
HFD-fed Transplanted mice
Biochemical detection
b
HFD-fed recipient (THKO)(LV-)
HFD-fed recipient (THKO)(LV+)
Liver weight (g)
Body weight (g)
Liver/body weight ratio (%)
ns.
d
Serum AST level (U/L)
Serum ALT level (U/L)
e
Blood glucose (mmol/L)
Time (min)
c
HFD-fed Recipient (THKO)(LV-)
HFD-fed Recipient (THKO)(LV+)
Liver appearance
H&E staining
Oil red O staining
PAS staining
f
Fasting blood glucose (mmol/L)
HOMA-IR (mIU/L×mmol/L)
(Weeks)
g
Liver TG contents (mg/g)
Liver TC contents (mg/g)
Liver NEFA contents (μmol/g)
h
Relative mRNA levels
HMGCR
SREBP-1c
CYP7A1
ABCG1
FATP1
FABP1
CD36
FAS
SCD1
ACCα
PPARγ
PDK4
PPARα
CPT-1α
ACOX-1
UCP2
LCAD
MCAD
Cholesterol synthesis & efflux
Fatty acid uptake
Fatty acid synthesis
Fatty acid oxidation
i
LV-
LV+
p-IRS1(Ser307)
p-IRS1(Tyr608)
IRS1
p-AKT
AKT
GAPDH
180 kDa
60 kDa
37 kDa
j
Rhbdf2
p-MAP3K7
MAP3K7
p-NF-κB
NF-κB
GAPDH
97 kDa
82 kDa
65 kDa
37 kDa
Relative protein levels (fold)
p-IRS1(Tyr608)/IRS1
p-IRS1(Ser307)/IRS1
p-AKT/AKT
Rhbdf2/GAPDH
p-MAP3K7/MAP3K7
p-NF-κB/NF-κB

Supplementary Figure 11. Allogeneic hepatocyte transplantation using lentivirus-mediated RING domain deletion of Trim31 did not alleviate HFD-triggered impaired insulin signaling and hepatic steatosis. Preconditioned liver-specific Trim31 deletion (THKO) mice with an 8-weeks HFD treatment as donor were transduced (THKO)(LV-). The hepatocytes isolated from (THKO)(LV-) group mice were transduced with lentivirus-loaded Trim31 with RING domain mutant sequence (Trim31 RING$^{\Delta}$). The corresponding blank vector were transduced as controls. Then, the additional HFD-fed THKO mice as recipient were injected with transduced hepatocytes via portal vein. The HFD-fed transplanted (THKO)(LV+) mice were harvested for the further experimental detection. (**a**) The strategy diagram for *ex vivo* gene therapy with LV- Trim31 RING$^{\Delta}$ used in the current study. (**b**) Records for the liver weight, body weight and the ratio of liver weight/body weight (%) of the HFD-fed recipient (THKO)(LV-) and (THKO)(LV+) mice at the last week of HFD treatment (*n*=25 mice per group) (*ns*., no significant differences compared with the HFD-fed recipient (THKO)(LV-) groups). (**c**) Representative pictures for liver appearance and Oil red O staining, H&E staining, and PAS staining-indicated liver histopathologic changes of the HFD-fed recipient (THKO)(LV-) and (THKO)(LV+) mice after *ex vivo* experiment (Magnification, 100×; *n*=10 images per group for each staining). (**d**) Liver function markers ALT and AST levels were detected in transduced recipient mice (*n*=25 mice per group) (*ns*., no significant differences compared with the HFD-fed recipient (THKO)(LV-) groups). (**e, f**) Records for the glucose tolerance test (GTT) (**E**) and insulin tolerance test (ITT) (**F**), and the corresponding fasting insulin levels and HOMA-IR index in the HFD-fed recipient (THKO)(LV-) and (THKO)(LV+) mice after transplantation (*n*=25 mice per group) (*ns*., no significant differences compared with the HFD-fed recipient (THKO)(LV-) groups). (**g**) Liver lipid contents including TG, TC and NEFA of the transduced mice (*n*=25 mice per group) (*ns*., no significant differences compared with the HFD-fed recipient (THKO)(LV-) groups). (**h**) qPCR analysis of the relative mRNA expression of genes associated with fatty acid uptake, synthesis, and β-oxidation in the HFD-fed recipient (THKO)(LV-) and (THKO)(LV+) mice after transplantation (*n*=10 liver samples per group) (*ns*., no significant differences compared with the HFD-fed recipient (THKO)(LV-) groups). (**i, j**) Representative immunoblotting bands for expression alterations of total amounts and phosphorylated forms of critical indicators involving in insulin signaling (**i**), including IRS1,

p-IRS1(Ser307), p-IRS1(Tyr608), AKT, and p-AKT, and Rhbdf2-MAP3K7 axis and p-NF-κB pathway (**j**) in the liver of HFD-fed recipient (THKO)(LV-) and (THKO)(LV+) mice (n=4 per experiment) (*ns*., no significant differences compared with the HFD-fed recipient (THKO)(LV-) groups). The GAPDH was used as loading control. Data are expressed as mean±SEM. The relevant experiments presented in this part were performed independently at least three times. Significance determined by Student's 2-tailed t-test analysis.

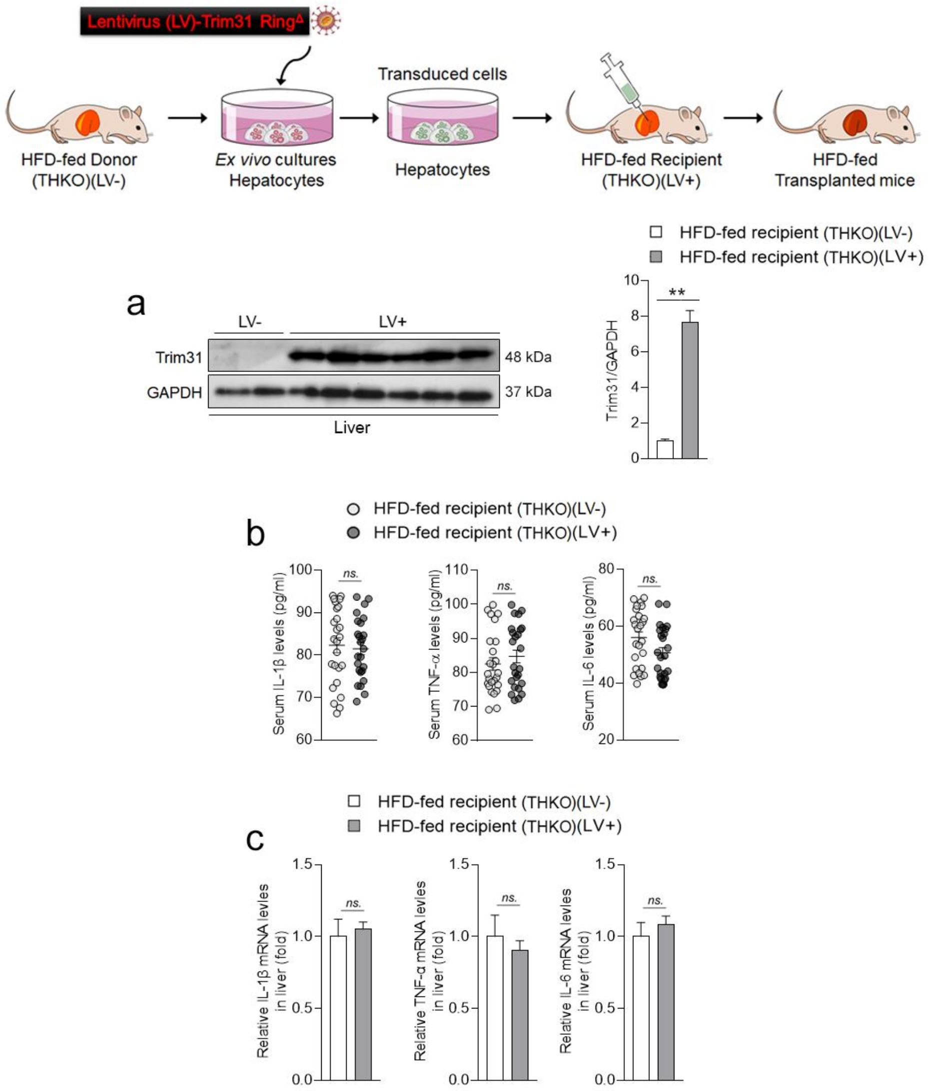
Lentivirus (LV)-Trim31 RingΔ
HFD-fed Donor (THKO)(LV-)
Ex vivo cultures Hepatocytes
Transduced cells
Hepatocytes
HFD-fed Recipient (THKO)(LV+)
HFD-fed Transplanted mice
a
LV-
LV+
Trim31
GAPDH
48 kDa
37 kDa
Liver
HFD-fed recipient (THKO)(LV-)
HFD-fed recipient (THKO)(LV+)
Trim31/GAPDH
**
b
Serum IL-1β levels (pg/ml)
Serum TNF-α levels (pg/ml)
Serum IL-6 levels (pg/ml)
ns.
c
Relative IL-1β mRNA levles in liver (fold)
Relative TNF-α mRNA levles in liver (fold)
Relative IL-6 mRNA levles in liver (fold)

Supplementary Figure 12. Allogeneic hepatocyte transplantation using lentivirus-mediated RING domain deletion of Trim31 fails to mitigate HFD-triggered liver inflammation. (**a**) Increased Trim31 expression in liver tissue after transplantation were detected by western blotting (*n*=6 per experiment) ($^{**}P < 0.01$ vs. HFD-fed transplanted (THKO)(LV-) groups). (**b**) Representative pro-inflammatory cytokines including TNF-α, IL-1β and IL-6 in serum from HFD-fed transplanted (THKO)(LV+) and HFD-fed transplanted (THKO)(LV-) mice after 16-weeks HFD feeding (*n*=25 mice per group) (*ns*., no significant differences compared with the HFD-fed recipient (THKO)(LV-) groups). (**c**) qPCR analysis of the relative mRNA expression of genes associated with inflammation signaling including TNF-α, IL-1β and IL-6 in the liver samples from HFD-fed transplanted (THKO)(LV+) and HFD-fed transplanted (THKO)(LV-) mice after 16-weeks HFD feeding (*n*=10 liver samples per group) (*ns*., no significant differences compared with the HFD-fed recipient (THKO)(LV-) groups). The GAPDH was used as loading control. Data are expressed as mean±SEM. The relevant experiments presented in this part were performed independently at least three times. Significance determined by Student's 2-tailed *t*-test analysis.

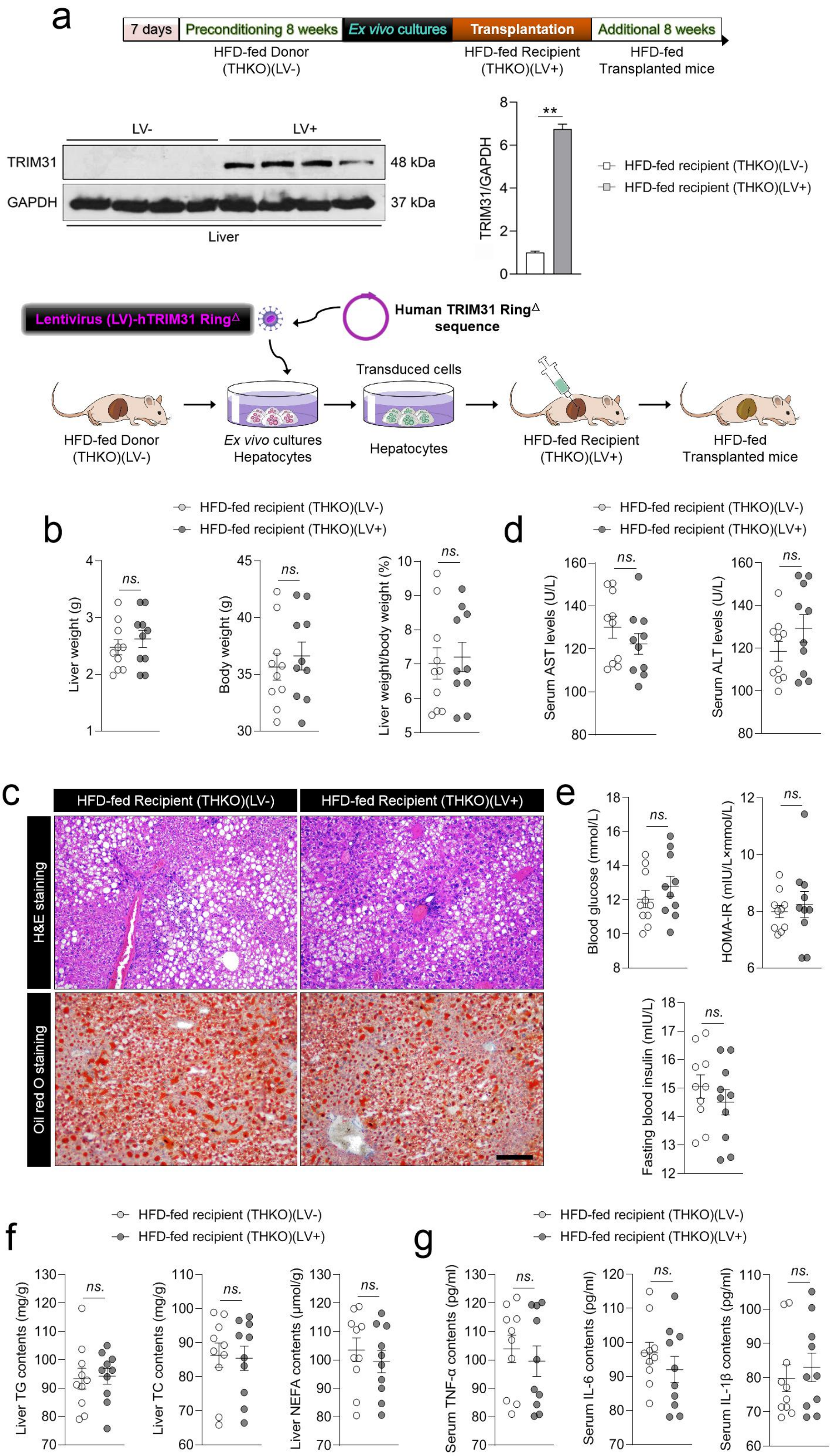
a
7 days
Preconditioning 8 weeks
Ex vivo cultures
Transplantation
Additional 8 weeks
HFD-fed Donor (THKO)(LV-)
HFD-fed Recipient (THKO)(LV+)
HFD-fed Transplanted mice
LV-
LV+
TRIM31
GAPDH
48 kDa
37 kDa
Liver
TRIM31/GAPDH
**
HFD-fed recipient (THKO)(LV-)
HFD-fed recipient (THKO)(LV+)
Lentivirus (LV)-hTRIM31 Ring△
Human TRIM31 Ring△ sequence
Transduced cells
HFD-fed Donor (THKO)(LV-)
Ex vivo cultures Hepatocytes
Hepatocytes
HFD-fed Recipient (THKO)(LV+)
HFD-fed Transplanted mice
b
d
Liver weight (g)
Body weight (g)
Liver weight/body weight (%)
Serum AST levels (U/L)
Serum ALT levels (U/L)
ns.
c
HFD-fed Recipient (THKO)(LV-)
HFD-fed Recipient (THKO)(LV+)
H&E staining
Oil red O staining
e
Blood glucose (mmol/L)
HOMA-IR (mIU/L×mmol/L)
Fasting blood insulin (mIU/L)
f
g
Liver TG contents (mg/g)
Liver TC contents (mg/g)
Liver NEFA contents (μmol/g)
Serum TNF-α contents (pg/ml)
Serum IL-6 contents (pg/ml)
Serum IL-1β contents (pg/ml)

Supplementary Figure 13. Lentivirus-mediated human TRIM31 (RING△) did not alleviate HFD-triggered impaired insulin signaling and hepatic steatosis. Preconditioned liver-specific Trim31 deletion (THKO) mice with an 8-weeks HFD treatment as donor were transduced (THKO)(LV-). The hepatocytes isolated from (THKO)(LV-) group mice were transduced with lentivirus-loaded human TRIM31 with RING domain mutant sequence (TRIM31 $RING^{\Delta}$). Then, the additional HFD-fed THKO mice as recipient were injected with transduced hepatocytes via portal vein. The HFD-fed transplanted (THKO)(LV+) mice were harvested for the further experimental detection. (**a**) The strategy diagram for *ex vivo* gene therapy with LV-TRIM31 $RING^{\Delta}$ used in the current study and representative western blotting bands for human TRIM31 expression in liver samples (*n*=6 per experiment) ($^{**}P < 0.01$ vs. HFD-fed transplanted (THKO)(LV-) groups). (**b**) Records for the liver weight, body weight and the ratio of liver weight/body weight (%) of the HFD-fed recipient (THKO)(LV-) and (THKO)(LV+) mice at the last week of HFD treatment (*n*=10 mice per group) (*ns*., no significant differences compared with the HFD-fed recipient (THKO)(LV-) groups). (**c**) Representative pictures for H&E staining and Oil red O staining-indicated liver histopathologic changes of the HFD-fed recipient (THKO)(LV-) and (THKO)(LV+) mice after *ex vivo* experiment (Magnification, 100×; *n*=10 images per group for each staining). (**d**) Liver function markers ALT and AST levels were detected in transduced recipient mice (*n*=10 mice per group) (*ns*., no significant differences compared with the HFD-fed recipient (THKO)(LV-) groups). (**e**) Records for the blood glucose, HOMA-IR index and fasting blood insulin levels in the HFD-fed recipient (THKO)(LV-) and (THKO)(LV+) mice after transplantation (*n*=10 mice per group) (*ns*., no significant differences compared with the HFD-fed recipient (THKO)(LV-) groups). (**f**) Liver lipid contents including TG, TC and NEFA of the transduced mice (*n*=10 mice per group) (*ns*., no significant differences compared with the HFD-fed recipient (THKO)(LV-) groups). (**g**) The pro-inflammatory cytokines levels (TNF-α, IL-6 and IL-1β) in serum (k) in the liver of HFD-fed recipient (THKO)(LV-) and (THKO)(LV+) mice (*n*=10 per experiment) (*ns*., no significant differences compared with the HFD-fed recipient (THKO)(LV-) groups). The GAPDH was used as loading control. Data are expressed as mean±SEM. The relevant experiments presented in this part were performed independently at least three times. Significance determined by Student's 2-tailed *t*-test analysis.

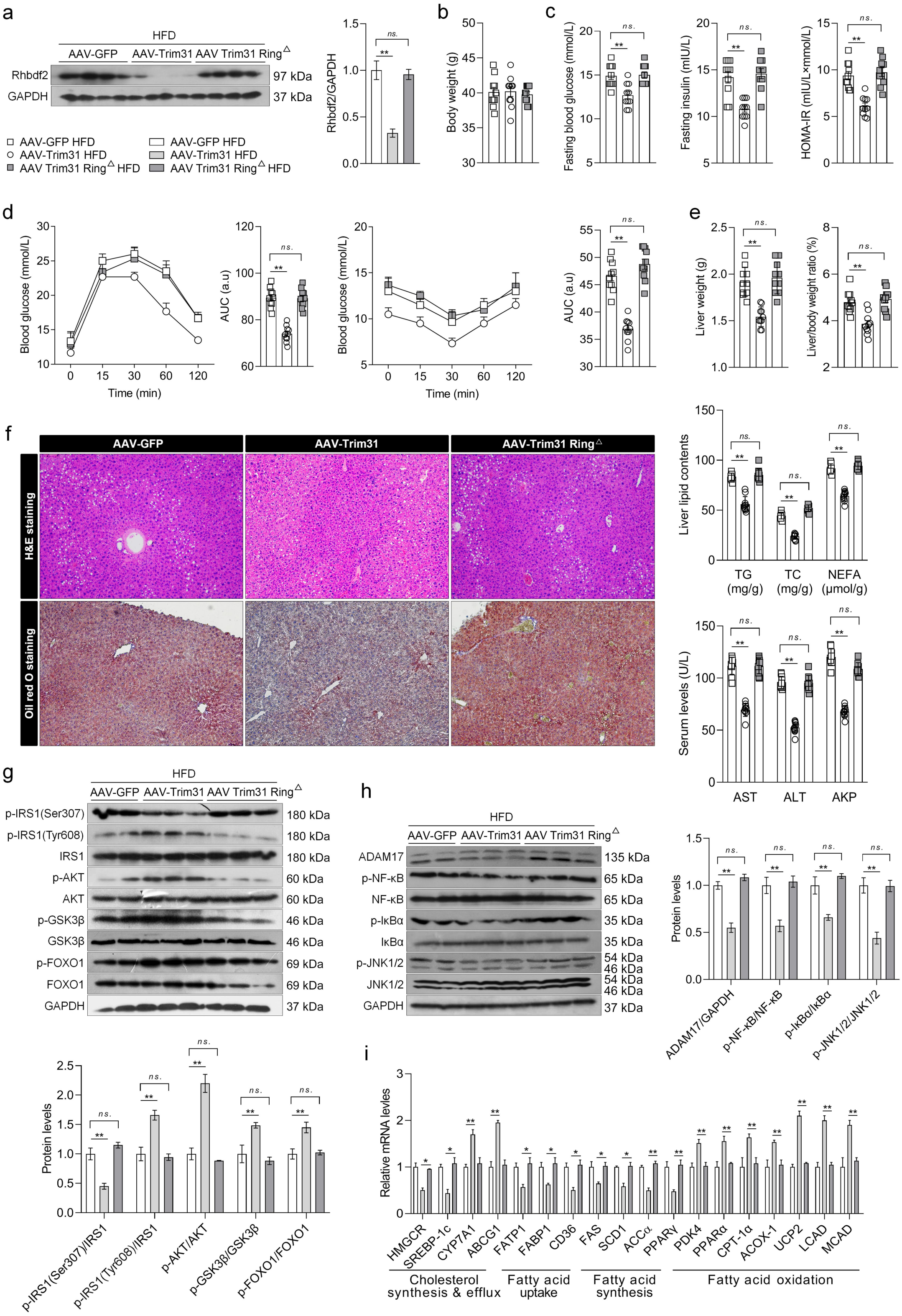

a
HFD
AAV-GFP
AAV-Trim31
AAV Trim31 Ring△
Rhbdf2
GAPDH
97 kDa
37 kDa
AAV-GFP HFD
AAV-Trim31 HFD
AAV Trim31 Ring△ HFD
Rhbdf2/GAPDH
b
Body weight (g)
c
Fasting blood glucose (mmol/L)
Fasting insulin (mIU/L)
HOMA-IR (mIU/L×mmol/L)
d
Blood glucose (mmol/L)
Time (min)
AUC (a.u)
e
Liver weight (g)
Liver/body weight ratio (%)
f
AAV-GFP
AAV-Trim31
AAV-Trim31 Ring△
H&E staining
Oil red O staining
Liver lipid contents
TG (mg/g)
TC (mg/g)
NEFA (μmol/g)
Serum levels (U/L)
AST
ALT
AKP
g
p-IRS1(Ser307)
p-IRS1(Tyr608)
IRS1
p-AKT
AKT
p-GSK3β
GSK3β
p-FOXO1
FOXO1
GAPDH
180 kDa
60 kDa
46 kDa
69 kDa
37 kDa
h
ADAM17
p-NF-κB
NF-κB
p-IκBα
IκBα
p-JNK1/2
JNK1/2
135 kDa
65 kDa
35 kDa
54 kDa
46 kDa
Protein levels
ADAM17/GAPDH
p-NF-κB/NF-κB
p-IκBα/IκBα
p-JNK1/2/JNK1/2
p-IRS1(Ser307)/IRS1
p-IRS1(Tyr608)/IRS1
p-AKT/AKT
p-GSK3β/GSK3β
p-FOXO1/FOXO1
i
Relative mRNA levels
HMGCR
SREBP-1c
CYP7A1
ABCG1
FATP1
FABP1
CD36
FAS
SCD1
ACCα
PPARγ
PDK4
PPARα
CPT-1α
ACOX-1
UCP2
LCAD
MCAD
Cholesterol synthesis & efflux
Fatty acid uptake
Fatty acid synthesis
Fatty acid oxidation

Supplementary Figure 14. RING-finger domain mutant of Trim31 fails to mediate Trim31-regualted hepatic steatosis, insulin resistance, and inflammation. To specifically over-expressed Trim31 in hepatocytes *in vivo* experiments, the adeno-associated virus serotype 8 (AAV8) encoding full-length Trim31 sequences (AAV-Trim31) and AAV8 encoding mutant Trim31 with RING domain deletion (AAV-Trim31 RING$^{\Delta}$) delivery system were established according to a standard molecular procedure. The 6-weeks HFD-fed preconditioned WT mice were injected with AAV-Trim31 or AAV-Trim31 RING$^{\Delta}$ by tail vein with 100 μl of virus containing 2×10^{11} vg of vectors and then fed with HFD for additional 10 weeks. The empty vector was injected into mice as the corresponding control. (**a**) Representative immunoblotting bands for Rhbdf2 expression alterations after AAV-Trim31 or AAV-Trim31 RING$^{\Delta}$ injection at the last week of HFD-fed WT mice (n=4 per experiment) ($^{**}P$ < 0.01 vs. AAV-GFP HFD groups, *ns*., no significant differences). (**b, c**) Records for the body weights (b), fasting blood glucose, fasting insulin levels and HOMA-IR index (c) of the AAV-injected WT mice at the last week of HFD treatment (n=10 mice per group) ($^{**}P$ < 0.01 vs. AAV-GFP HFD groups, *ns*., no significant differences). (**d**) Records for the glucose tolerance test (GTT) and insulin tolerance test (ITT) in the HFD-fed indicated mice after AAV injection (n=10 mice per group) ($^{**}P$ < 0.01 vs. AAV-GFP HFD groups, *ns*., no significant differences). (**e**) Records for the liver weight, the ratio of liver weight/body weight (%), hepatic lipid contents (TG, TC and NEFA) and liver function indicators including AST, ALT and AKP levels of the HFD-fed indicated mice after AAV injection (n=10 mice per group) ($^{**}P$ < 0.01 vs. AAV-GFP HFD groups, *ns*., no significant differences). (**f**) Representative images for H&E staining, Oil red O staining-indicated liver histopathologic changes, lipid contents and liver function-related indexes of the HFD-fed mice after AAV injection (Magnification, 100×; n=10 images per group for each staining) ($^{**}P$ < 0.01 vs. AAV-GFP HFD groups, *ns*., no significant differences) ($^{**}P$ < 0.01 vs. AAV-GFP HFD groups, *ns*., no significant differences). (**g**) Representative immunoblotting bands for expression alterations of total amounts and phosphorylated forms of critical indicators involving in insulin signaling, including IRS1, AKT, GSK3β and FOXO1, in the liver of mice with AAV-Trim31 or AAV-Trim31 RING$^{\Delta}$ injection after HFD feeding for 16 weeks (n=4 per experiment) ($^{**}P$ < 0.01 vs. AAV-GFP HFD groups, *ns*., no significant differences). (**h**) Representative immunoblotting bands for expression alterations of

total amounts and phosphorylated forms of critical indicators associating with inflammation signaling, including ADAM17, IκBα, p-IκBα, JNK1/2, p-JNK1/2, NF-κB and p-NF-κB, in the liver of mice with AAV-Trim31 or AAV-Trim31 RING$^{\Delta}$ injection after HFD feeding for 16 weeks (*n*=4 per experiment) ($^{**}P < 0.01$ vs. AAV-GFP HFD groups, *ns*., no significant differences). (**i**) qPCR analysis of the relative mRNA expression of genes associated with fatty acid uptake, synthesis, and β-oxidation in the HFD-fed indicated mice after AAV injection (*n*=10 liver samples per group) ($^{*}P < 0.05$ and $^{**}P < 0.01$ vs. AAV-GFP HFD groups, *ns*., no significant differences). The GAPDH was used as loading control. Data are expressed as mean±SEM. The relevant experiments presented in this part were performed independently at least three times. Significance determined by One-way analysis of variance (ANOVA) followed by Dunnett's multiple comparisons test.

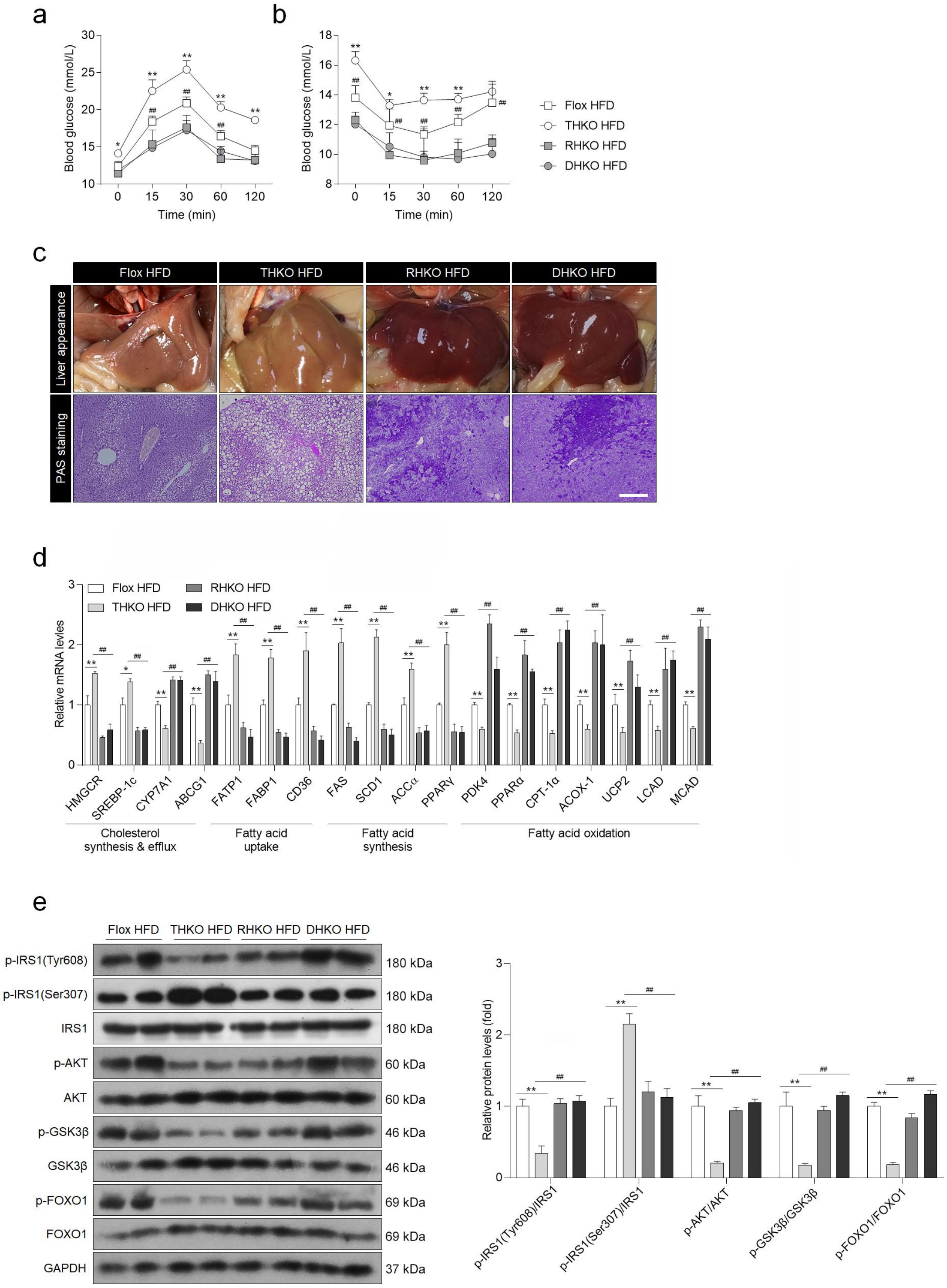
a
Blood glucose (mmol/L)
Time (min)
b
Blood glucose (mmol/L)
Time (min)
Flox HFD
THKO HFD
RHKO HFD
DHKO HFD
c
Flox HFD
THKO HFD
RHKO HFD
DHKO HFD
Liver appearance
PAS staining
d
Flox HFD
THKO HFD
RHKO HFD
DHKO HFD
Relative mRNA levles
HMGCR
SREBP-1c
CYP7A1
ABCG1
FATP1
FABP1
CD36
FAS
SCD1
ACCα
PPARγ
PDK4
PPARα
CPT-1α
ACOX-1
UCP2
LCAD
MCAD
Cholesterol synthesis & efflux
Fatty acid uptake
Fatty acid synthesis
Fatty acid oxidation
e
Flox HFD
THKO HFD
RHKO HFD
DHKO HFD
p-IRS1(Tyr608)
p-IRS1(Ser307)
IRS1
p-AKT
AKT
p-GSK3β
GSK3β
p-FOXO1
FOXO1
GAPDH
180 kDa
180 kDa
180 kDa
60 kDa
60 kDa
46 kDa
46 kDa
69 kDa
69 kDa
37 kDa
Relative protein levels (fold)
p-IRS1(Tyr608)/IRS1
p-IRS1(Ser307)/IRS1
p-AKT/AKT
p-GSK3β/GSK3β
p-FOXO1/FOXO1

Supplementary Figure 15. Hepatocyte-specific Trim31 and Rhbdf2 double deletion mitigates HFD-induced insulin resistance. (**a, b**) Records for the glucose tolerance test (GTT) (a) and insulin tolerance test (ITT) (b) in the 16-weeks HFD-fed mice (*n*=10 mice per group) ($^{*}P < 0.05$ and $^{**}P < 0.01$ vs. Flox HFD groups, $^{\#\#}P < 0.01$ vs. THKO HFD groups). (**c**) Representative images for liver appearance and PAS staining-indicated liver histopathologic changes of the HFD-fed mice (Magnification, 100×; *n*=10 images per group for each staining). (**d**) qPCR analysis of the relative mRNA expression of genes associated with fatty acid uptake, synthesis, and β-oxidation in the HFD-fed indicated mice after HFD treatment (*n*=10 liver samples per group) ($^{*}P < 0.05$ and $^{**}P < 0.01$ vs. Flox HFD groups, $^{\#\#}P < 0.01$ vs. THKO HFD groups). (**e**) Representative immunoblotting bands for expression alterations of total amounts and phosphorylated forms of critical indicators involving in insulin signaling, including IRS1, AKT, GSK3β and FOXO1, in the liver of mice after HFD feeding for 16 weeks (*n*=4 per experiment) ($^{**}P < 0.01$ vs. Flox HFD groups, $^{\#\#}P < 0.01$ vs. THKO HFD groups). The GAPDH was used as loading control. Data are expressed as mean±SEM. The relevant experiments presented in this part were performed independently at least three times. Significance determined by two-way analysis of variance with general linear model procedures using a univariate approach.

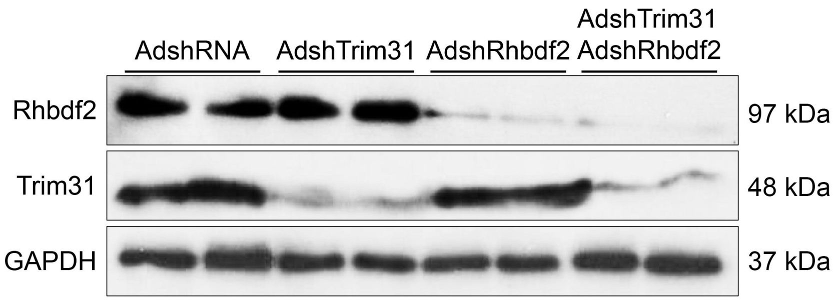

Supplementary Figure 16. The transfection efficiency of adenovirus-induced hepatocytes. Representative western blotting bands of the primary hepatocytes that were transfected with AdshRhbdf2 or AdshTrim31 or co-treated with AdshRhbdf2/AdshTrim31 (*n*=4 per experiment).

a

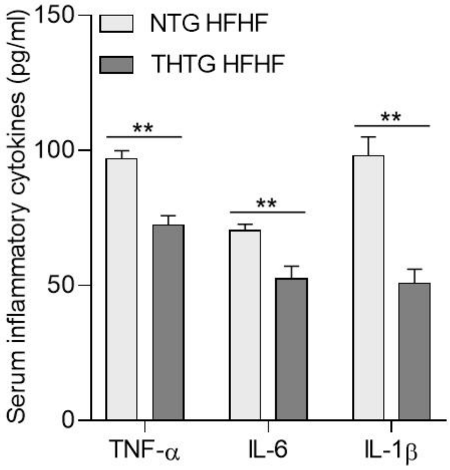

NTG HFHF
THTG HFHF
Serum inflammatory cytokines (pg/ml)
150
100
50
0
**
**
**
TNF-α
IL-6
IL-1β

b

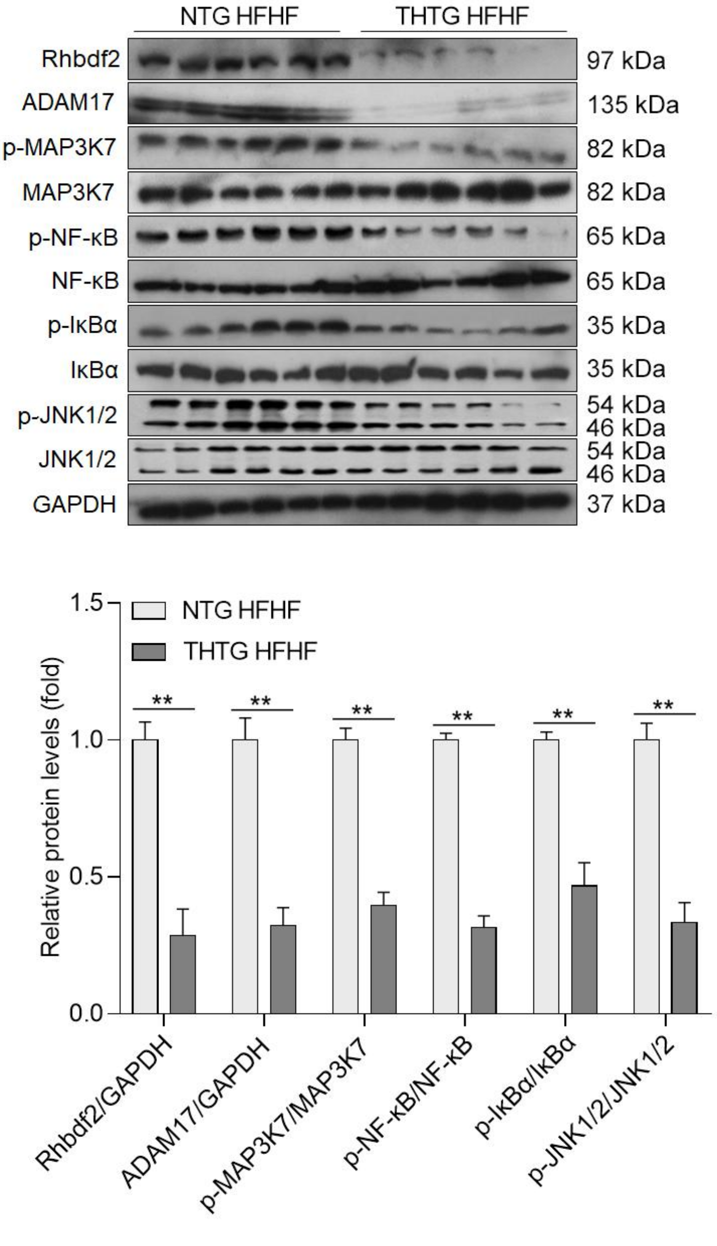

NTG HFHF
THTG HFHF
Rhbdf2
97 kDa
ADAM17
135 kDa
p-MAP3K7
82 kDa
MAP3K7
82 kDa
p-NF-κB
65 kDa
NF-κB
65 kDa
p-IκBα
35 kDa
IκBα
35 kDa
p-JNK1/2
54 kDa
46 kDa
JNK1/2
54 kDa
46 kDa
GAPDH
37 kDa
NTG HFHF
THTG HFHF
Relative protein levels (fold)
1.5
1.0
0.5
0.0
**
**
**
**
**
**
Rhbdf2/GAPDH
ADAM17/GAPDH
p-MAP3K7/MAP3K7
p-NF-κB/NF-κB
p-IκBα/IκBα
p-JNK1/2/JNK1/2

Supplementary Figure 17. Trim31 overexpression suppresses Rhbdf2/MAP3K7 axis-associated liver inflammation in HFHF-induced NASH model. (**a**) Representative pro-inflammatory cytokines including TNF-α, IL-1β and IL-6 in serum from HFHF-fed NTG- and THTG mice (*n*= 15 mice per group) ($^{**}P$ < 0.01 vs. NTG HFHF groups). (**b**) Representative immunoblotting bands for expression alterations of total amounts and phosphorylated forms of critical indicators involving in inflammation signaling, including Rhbdf2-MAP3K7 and its downstream events cascades, in the liver of mice after HFHF feeding for 16 weeks (*n*=6 per experiment) ($^{**}P$ < 0.01 vs. NTG HFHF groups). The GAPDH was used as loading control. Data are expressed as mean±SEM. The relevant experiments presented in this part were performed independently at least three times. Significance determined by Student's 2-tailed *t*-test analysis.

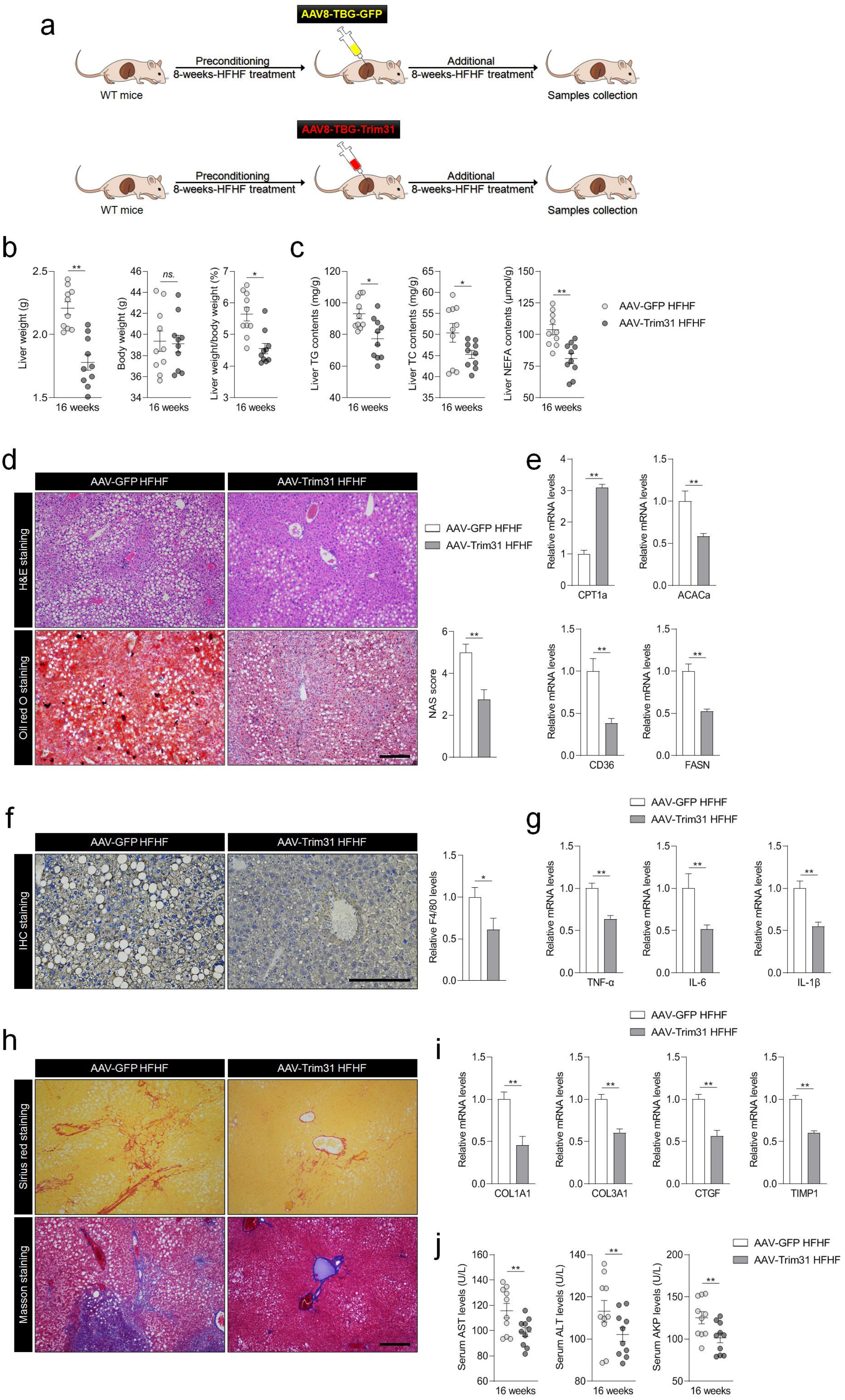
a
AAV8-TBG-GFP
Preconditioning
8-weeks-HFHF treatment
Additional
8-weeks-HFHF treatment
WT mice
Samples collection
AAV8-TBG-Trim31
b
Liver weight (g)
Body weight (g)
Liver weight/body weight (%)
16 weeks
c
Liver TG contents (mg/g)
Liver TC contents (mg/g)
Liver NEFA contents (μmol/g)
AAV-GFP HFHF
AAV-Trim31 HFHF
d
H&E staining
Oil red O staining
NAS score
e
Relative mRNA levels
CPT1a
ACACa
CD36
FASN
f
IHC staining
Relative F4/80 levels
g
TNF-α
IL-6
IL-1β
h
Sirius red staining
Masson staining
i
COL1A1
COL3A1
CTGF
TIMP1
j
Serum AST levels (U/L)
Serum ALT levels (U/L)
Serum AKP levels (U/L)

Supplementary Figure 18. AAV8-mediated Trim31 gene therapy protects against HFHF-induced nonalcoholic steatohepatitis (NASH) *in vivo.* (**a**) An experiment design of AAV8-mediated Trim31 gene therapy in HFHF-induced NASH mouse model. (**b**) Records for liver weight, body weight and ratio of liver weight/body weight in AAV-GFP and AAV-Trim3 groups after a 16-weeks HFHF administration (*n*=10 mice per group) ($^{*}P < 0.05$ and $^{**}P < 0.01$ vs. AAV-GFP HFHF groups, *ns*., no significant difference). (**c**) Liver lipid contents including TG, TC and NEFA of the indicated mice after a 16-weeks HFHF ingestion (*n*=10 mice per group) ($^{*}P < 0.05$ and $^{**}P < 0.01$ vs. AAV-GFP HFHF groups). (**d**) Representative pictures for H&E staining and Oil red O staining-indicated liver histopathologic changes of a 16-weeks HFHF-fed mice (Magnification, 100×; *n*=5 images per group for each staining) ($^{**}P < 0.01$ vs. AAV-GFP HFHF groups). (**e**) qPCR analysis of the relative mRNA expression of genes in the livers associated with fatty acid metabolism after a 16-weeks HFHF administration (*n*=10 liver samples per group) ($^{**}P < 0.01$ vs. AAV-GFP HFHF groups). (**f**) Immunohistochemistry staining of F4/80 analysis showing the histopathologic changes of the liver samples in indicated mice (Magnification, 200×; *n*=5 images per group for each staining) ($^{*}P < 0.05$ vs. AAV-GFP HFHF groups). (**g**) qPCR analysis of the relative mRNA expression of genes in the livers associated with inflammation after a 16-weeks HFHF administration (*n*=10 liver samples per group) ($^{**}P < 0.01$ vs. AAV-GFP HFHF groups). (**h**) Representative pictures for Sirius red staining and Masson staining showing the collagen deposition levels of the liver samples in indicated mice (Magnification, 100×; *n*=5 images per group for each staining). (**i, j**) qPCR analysis of the relative mRNA expression of genes in the livers associated with collagen synthesis after a 16-weeks HFHF administration (i) (*n*=10 liver samples per group); and hepatic function indicator AST, ALT and AKP levels in the 16-weeks HFHF-fed AAV-GFP or AAV-Trim3 injected mice (j) (*n*=10 mice per group) ($^{**}P < 0.01$ vs. AAV-GFP HFHF groups). Data are expressed as mean±SEM. The relevant experiments presented in this part were performed independently at least three times. Significance determined by Student's 2-tailed *t*-test analysis.

Supplementary Table 1. The clinical information of non-steatosis, simple steatosis and NASH patients.

Items	Sex	Age (Years)	BMI (Kg/m^2)	AST (U/L)	ALT (U/L)	TG (mmol/L)	TC (mmol/L)	Fasting blood glucose (mmol/L)	Serum TNF-α (ng/ml)	Serum IL-6 (pg/ml)	NAS
Non-steatosis	Male/Female (11/5)	35.1 (21-47)	19.6 (17.9-21.6)	24.8 (15.6-30.7)	21.9 (10.7-27.5)	1.7 (1.47-1.6)	3.8 (3.73-4.78)	5.0 (4.1-6.1)	9.9 (8.62-11.37)	33.5 (24.51-52.16)	0
Simple steatosis	Male/Female (10/7)	35.7 (32-51)	25.2 (24.6-26.1)	54.2 (41.5-62.5)	45.5 (30.7-57.7)	2.1 (1.78-2.5)	4.2 (3.33-5.10)	5.7 (4.8-7.1)	13.4 (8.54-18.70)	52.9 (35.28-74.54)	1.7
NASH	Male/Female (8/8)	35.9 (22-55)	30.1 (24.4-37.4)	51.9 (36.6-64.4)	42.1 (31.8-57.2)	2.3 (1.67-3.54)	4.4 (3.12-5.96)	6.2 (5.0-7.9)	21.2 (13.09-28.29)	55.9 (36.63-82.28)	4.3

Data were expressed as mean for all items. The values in bracket indicate the minimum and maximum value in corresponding item.

Supplementary Table 2. The primer sequences for the targeted genes used in this study.

Primers	Forward Sequence (5'-3')	Reverse Sequence (5'-3')
Rhbdf2	CACAGCCCAGTGGTTTGGGGTCA	GAGGGCGGCGGCTGCCTGAAAGCT
Trim31	CCAGAGTCAAACCGTGAGCG	GGCAACTTGGAGCCCGAA
TNFR1	TACATTGCAGCCTCTGCCTC	AGAGCTTGGACTTCCACCGT
TNFR2	ACATCAGACGTGGTGGTGCAA	CCAACTGGAAGAGCCAAGTC
TNF-α	ACCTGGCCTCTCTACCTTGT	CCCGTAGGGCGATTACAGTC
IL-1β	AGCAAAGTGATAGGCCGAC	GCGTCGCAATTAGTGTTGTG
Il-6	GGGCAACAAGATTCCGATATA	AGCCACTACATGGAATCTAAT
MCP-1	CTGGTCCGAGTGAGACAAAG	AGATCAGGCTCTGATGGAGAA
iNOS	CCAACCGGAGAAGGGGACGAACT	GGAGGGTGGTGCGGCTGGAC
CXCR1	GCTGCCCACTGGAGATTATTTC	TATGCCTGGCGGAAGATAGC
CCL1	GGCTGAACAAAGGTAGAGAAAGC	TGGAGGACTGAGGGAAACTG
IL-8	ATGACTTCCAAGCTGGCCGTGGCT	TCTCAGCCCTCTTCAAAAACTTCTC
FKN	ACCTATGGCCCTGACATCATCAC	CTTGCCAGCCCTCAGAATCAC
MDC	TCTGATGCAGGTCCCTATGGT	TTATGGAGTAGCTTCTTCAC
MIP-1	CCCGACTGCCTGCTGCTTCTCC	CAAAGGCTGCTGGTCTCAAA
COX-2	CCACCTCTGCGATGCTCTTC	ACATTCCCCACGGTTTTGAC
CX3CR1	CCGGTCTCATTTGCAGGCTTA	TGGGAATTGAACTTGGGACCTC
IL-18	CAAGGCTGGTCCATGCTCC	TGCTATCACTTCCTTTCTGTTGC
CXCR4	GAAGCTCTTGGTGAAAAGG	GAGTCGATGCTGATCCCAAT
CCR2	CCTTGGGAATGAGTAACTGTGTGA	ACAAAGGCATAAATGACAGGATTAATG
CXCL2	CGGTCAAAAAGTTTGCCTTG	TCCAGGTCAGTTAGCCTTGC
ICAM1	TAAGAGGACTCGGTGGATGG	ATGAGGCTCGATTGTTCAGC
Col4	TGTCATGGTGTGAAGGGACA	TCTCCAGCATCACCCTTTTG
SREBP-1c	AGGGGTAGGGCCAACGGCCT	CAAGCTGCCTGGGGAGCTGGTA
HMGCR	TGTCCTTGATGGCAGCCTTG	CGCGCTTCAGTTCAGTGTCAG
ABCG1	GATTGGCTTCAGGATGTCCATGTTGGAA	GTATTTTTGCAAGGCTACCAGTTACATTTGACAA
UCP2	GTTCCTCTGTCTCGTCTTGC	GGCCTTGAAACCAACCA
CYP7A1	CTGTGTTCACTTTCTGAAGCCATG	CCCAGGCATTGCTCTTTGAT
FATP1	GATGTGCTCTACGACTGTCTG	CAGCCCATAGATGAGACACTG
FABP1	GCAGAGCCAGGAGAACTTTG	GGGTCCATAGGTGATGGTGAG
CD36	AGCAACTGGTGGATGGTTTC	TCAAGGGAGAGCACTGGTTT
CPT-1α	TCTTGCAGTCGACTCACCTT	TCCACAGGACACATAGTCAGG
PPARα	CAAGGCCTCAGGGTACCACT	TTGCAGCTCCGATCACACTT
PPAR-γ	CAAGACTACCCTTTACTGAA	CTACTTTGATCGCACTTTGGT
ACCα	GGCCAGTGCTATGCTGAGAT	AGGGTCAAGTGCTGCTCCA
FAS	CTGCGGAAACTTCAGGAAATG	GGTTCGGAATGCTATCCAGG
SCD1	CTTCTTGCGATACACTCTGG	TGAATGTTCTTGTCGTAGGG
PDK4	TGTGATGTGGTAGCAGTAGTC	GTGGTGAAGGTGTGAAGGA

ACOX-1	GAATTTGGCATCGCAGACCC	GATCTCCAGATTCCAGGCCG
LCAD	AAGGATTTATTAAGGGCAAGAAGC	GGAAGCGGAGGCGGAGTC
MCAD	GAGCCTGGGAACTCGGCTTGA	GCCAAGGCCACCGCAACTTT
GAPDH	GGTGAAGGTCGGTGTGAACG	CCCGTAGGGCGATTACAGTC

Uncropped western blotting bands used in this study

Figure 1a

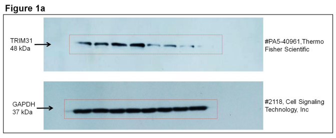

Figure 1b

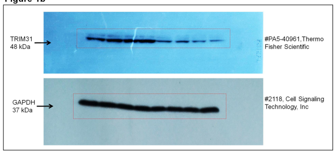

Figure 1c

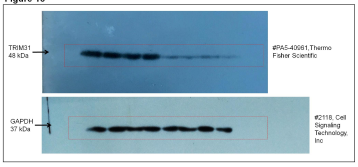

Figure 1d

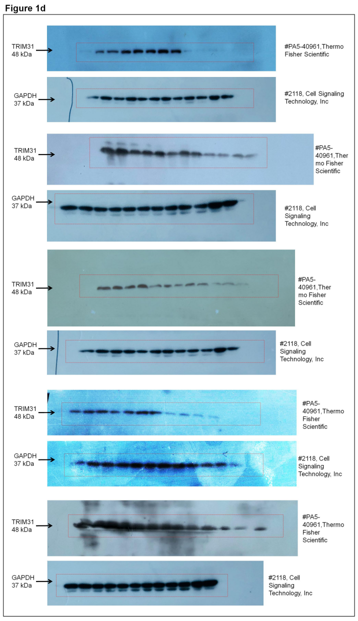

Figure 1e

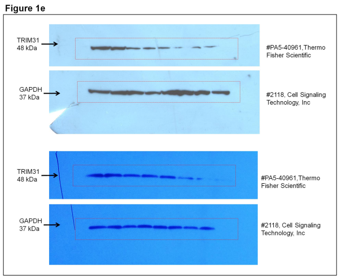

Figure 1f

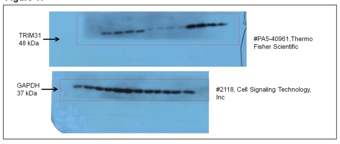

Figure 1g

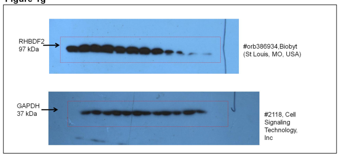

Figure 1h

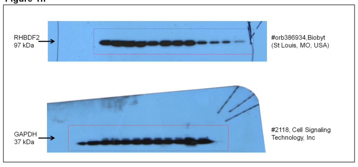

Figure 1i

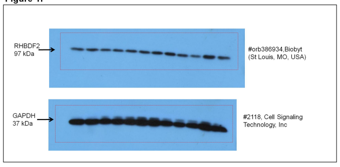

Figure 2h

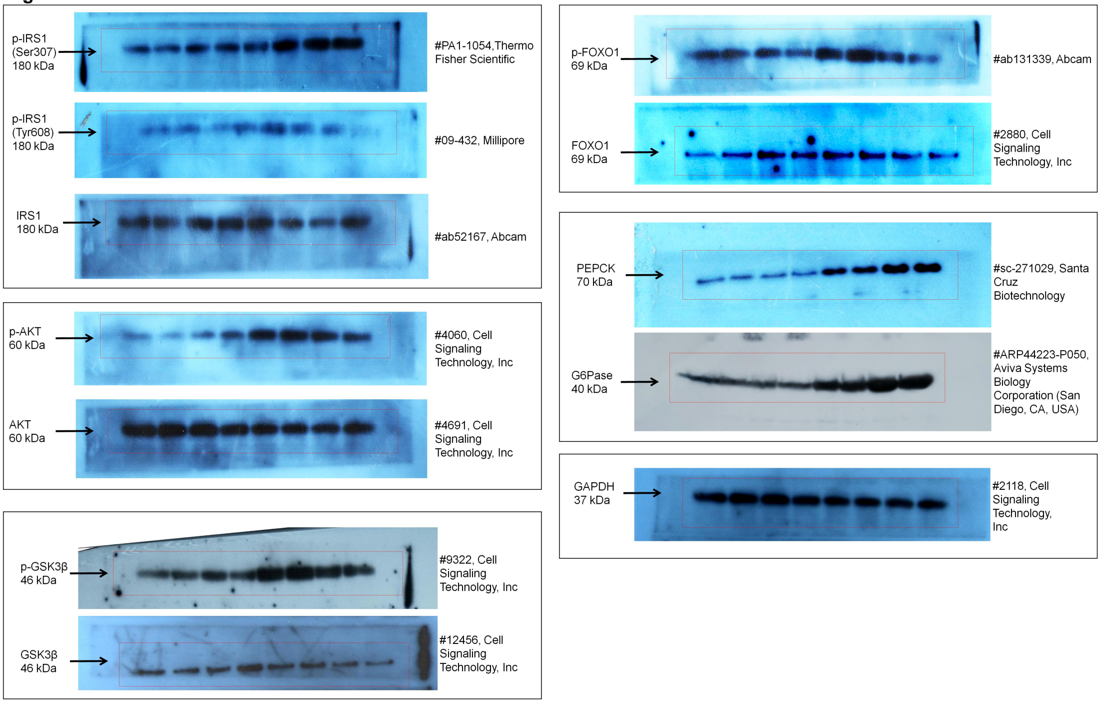

Figure 4i

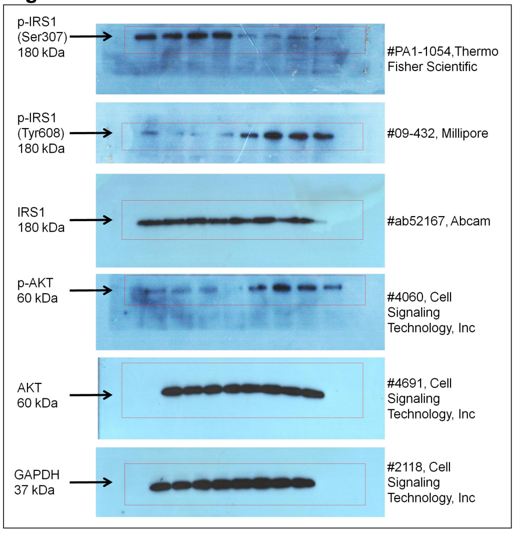

Figure 4j

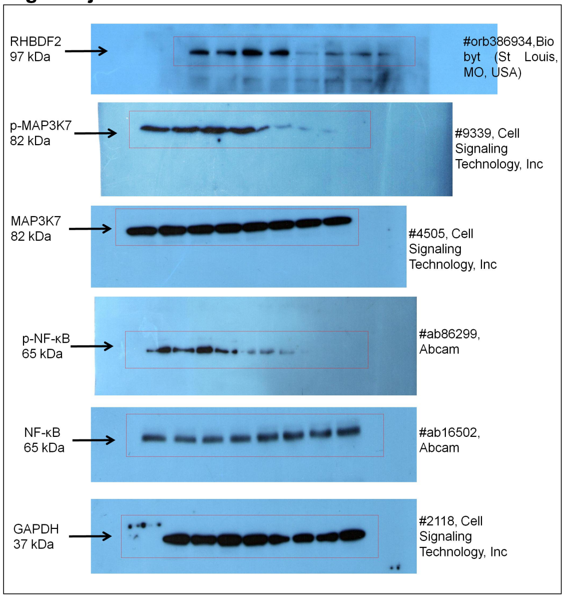

Figure 5b

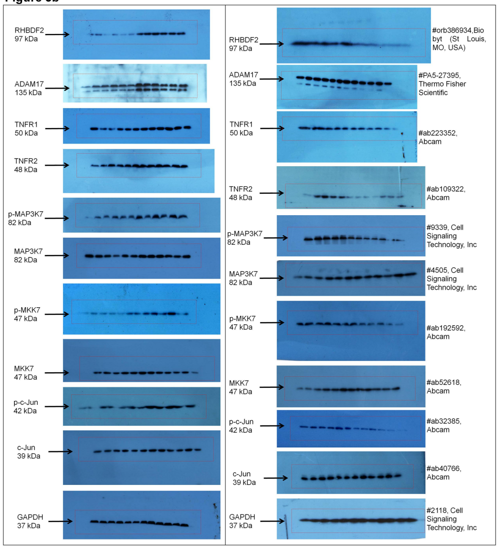

Figure 5c

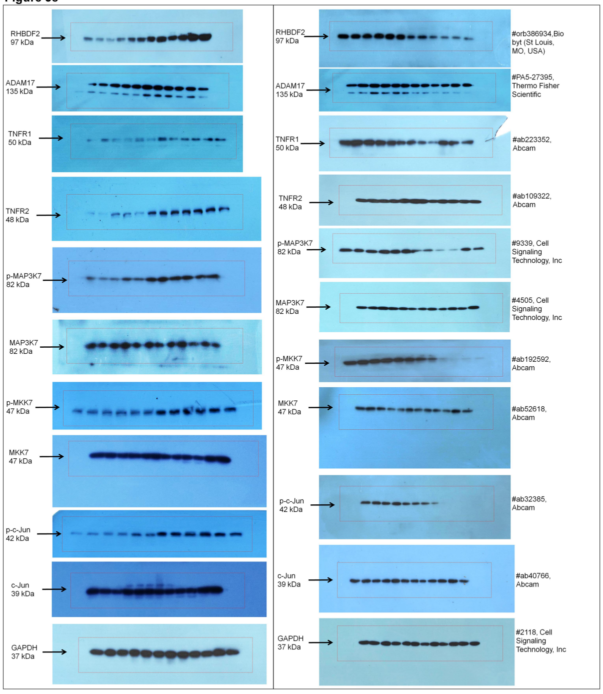

Figure 5d

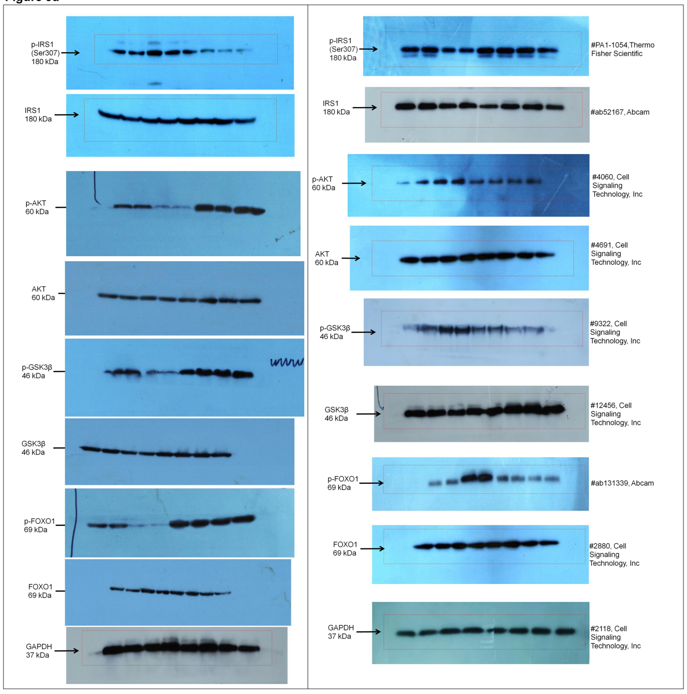

Figure 6

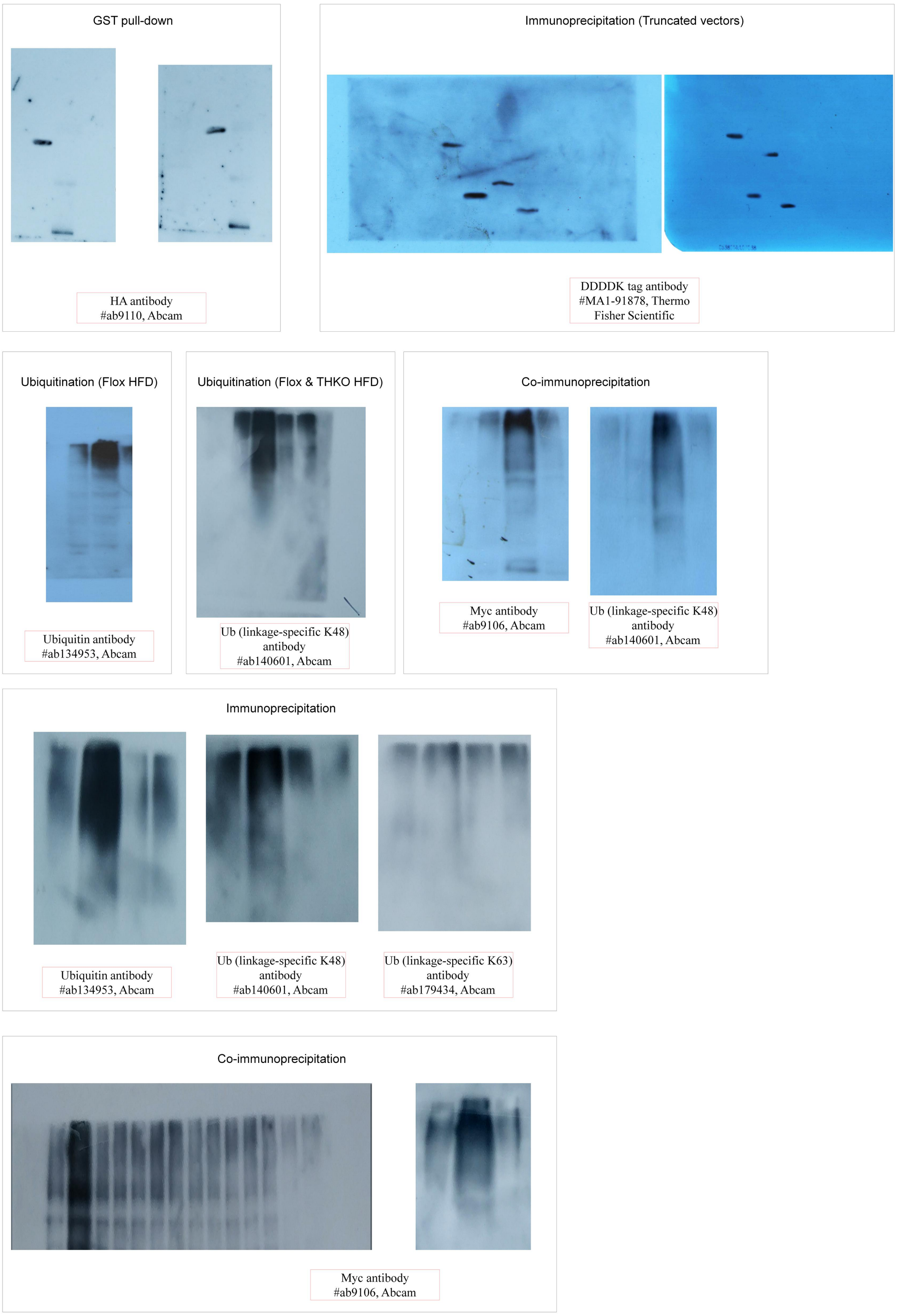
GST pull-down
HA antibody
#ab9110, Abcam
Immunoprecipitation (Truncated vectors)
DDDDK tag antibody
#MA1-91878, Thermo
Fisher Scientific
Ubiquitination (Flox HFD)
Ubiquitin antibody
#ab134953, Abcam
Ubiquitination (Flox & THKO HFD)
Ub (linkage-specific K48)
antibody
#ab140601, Abcam
Co-immunoprecipitation
Myc antibody
#ab9106, Abcam
Ub (linkage-specific K48)
antibody
#ab140601, Abcam
Immunoprecipitation
Ubiquitin antibody
#ab134953, Abcam
Ub (linkage-specific K48)
antibody
#ab140601, Abcam
Ub (linkage-specific K63)
antibody
#ab179434, Abcam
Co-immunoprecipitation
Myc antibody
#ab9106, Abcam

Figure 7a

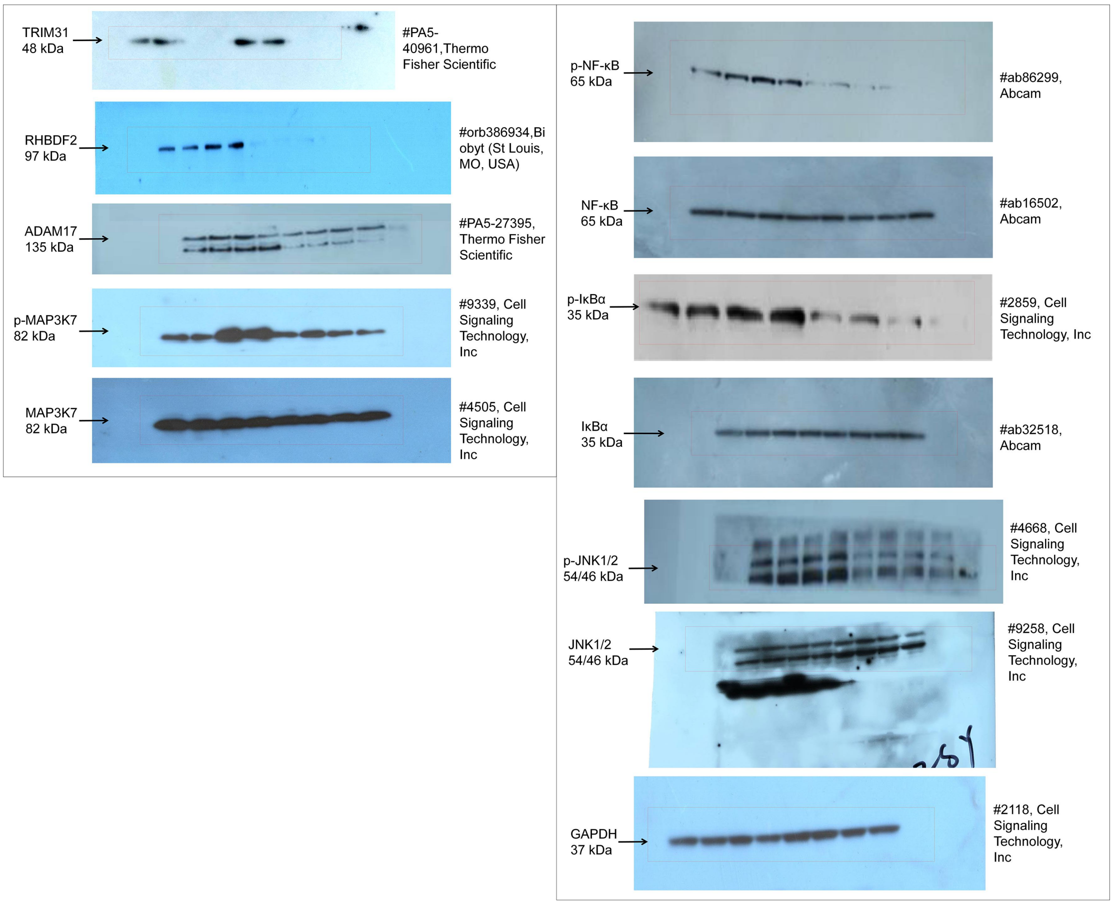

TRIM31
48 kDa
#PA5-40961,Thermo Fisher Scientific
RHBDF2
97 kDa
#orb386934,Biobyt (St Louis, MO, USA)
ADAM17
135 kDa
#PA5-27395, Thermo Fisher Scientific
p-MAP3K7
82 kDa
#9339, Cell Signaling Technology, Inc
MAP3K7
82 kDa
#4505, Cell Signaling Technology, Inc
p-NF-κB
65 kDa
#ab86299, Abcam
NF-κB
65 kDa
#ab16502, Abcam
p-IκBα
35 kDa
#2859, Cell Signaling Technology, Inc
IκBα
35 kDa
#ab32518, Abcam
p-JNK1/2
54/46 kDa
#4668, Cell Signaling Technology, Inc
JNK1/2
54/46 kDa
#9258, Cell Signaling Technology, Inc
GAPDH
37 kDa
#2118, Cell Signaling Technology, Inc

Supplementary Figure 1a

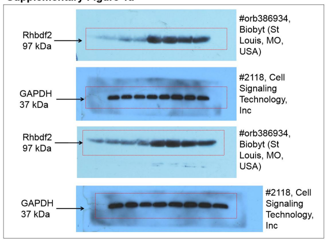

Supplementary Figure 1e

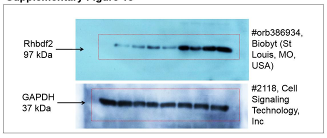

Supplementary Figure 1f

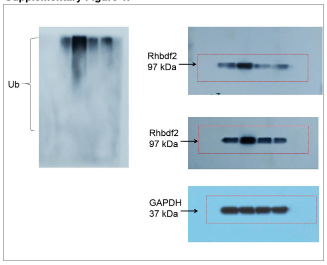

Supplementary Figure 1d

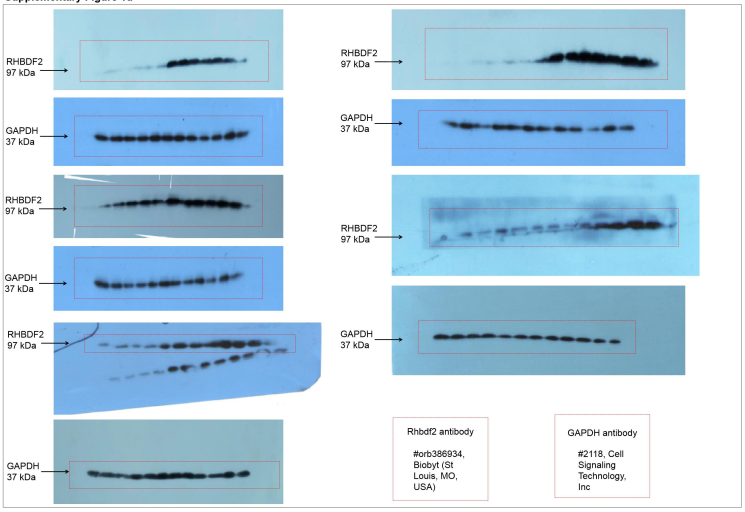

Supplementary Figure 3b

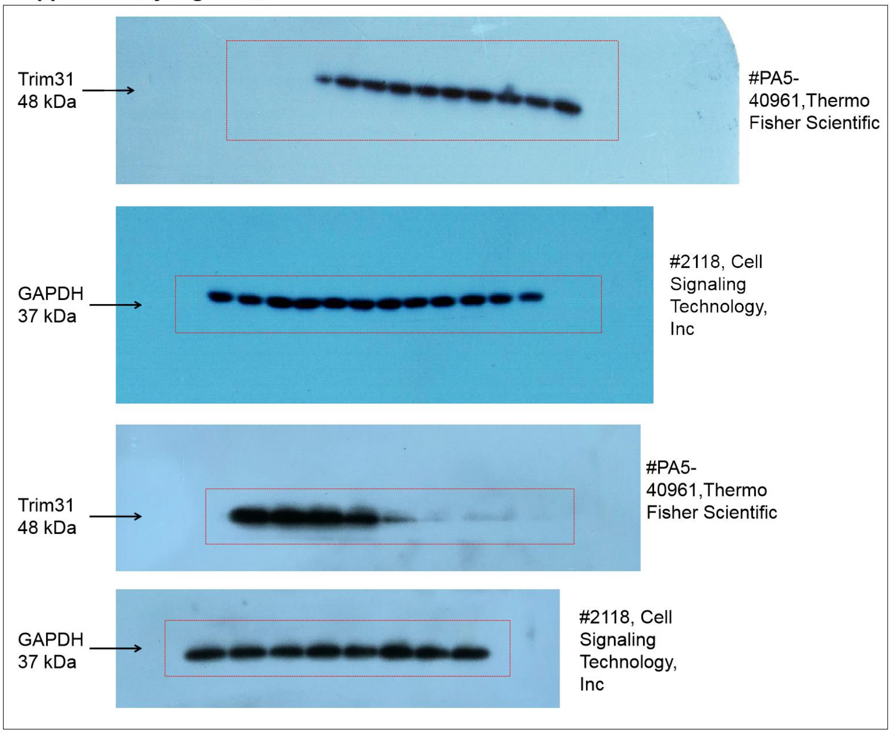

Supplementary Figure 4b

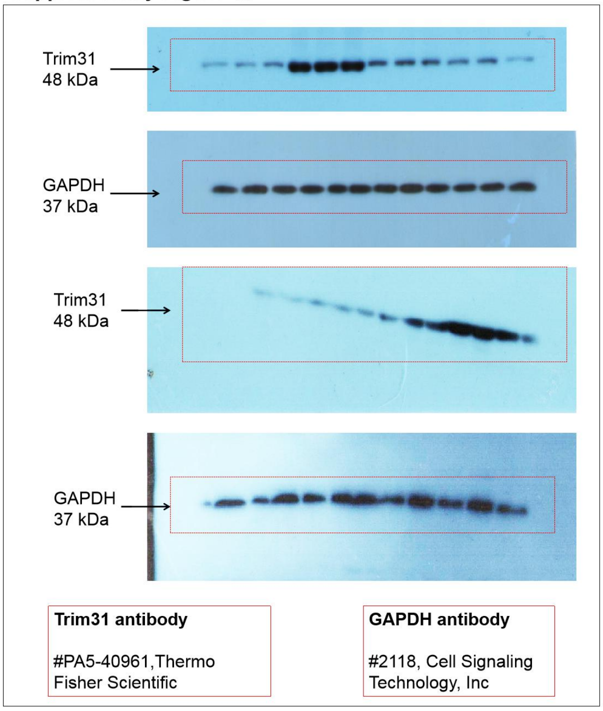

Supplementary Figure 4d

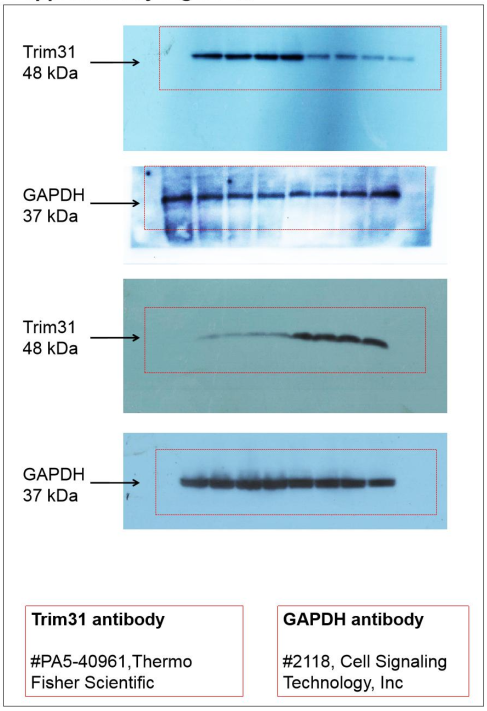

Supplementary Figure 5h

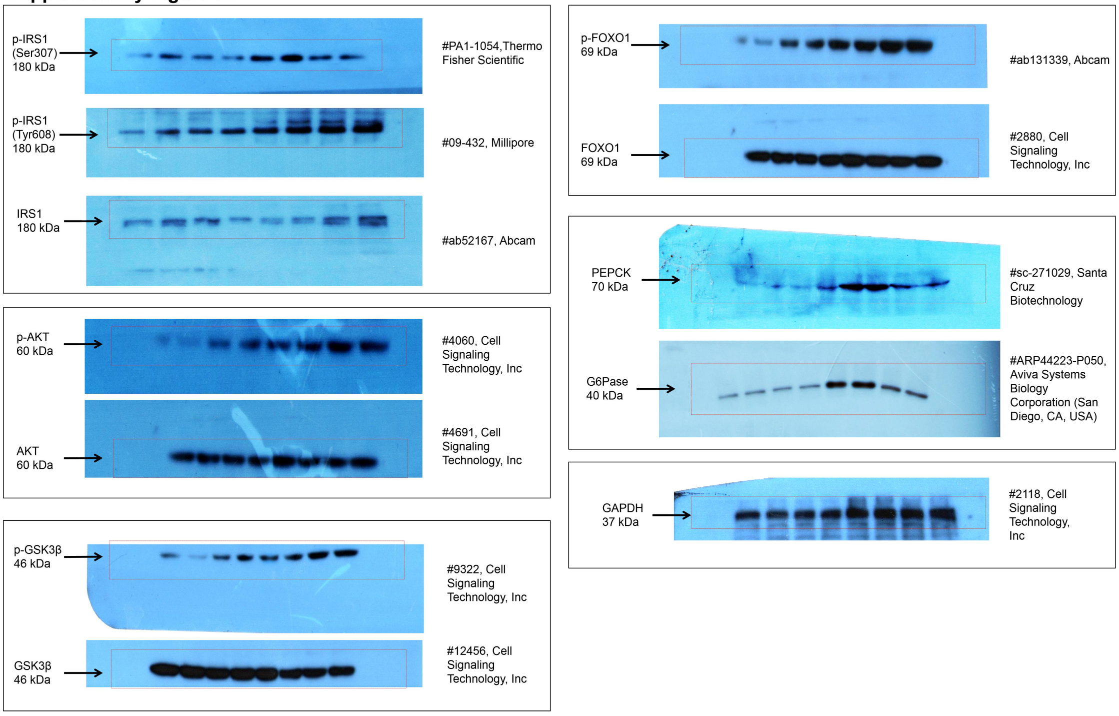

Supplementary Figure 7a

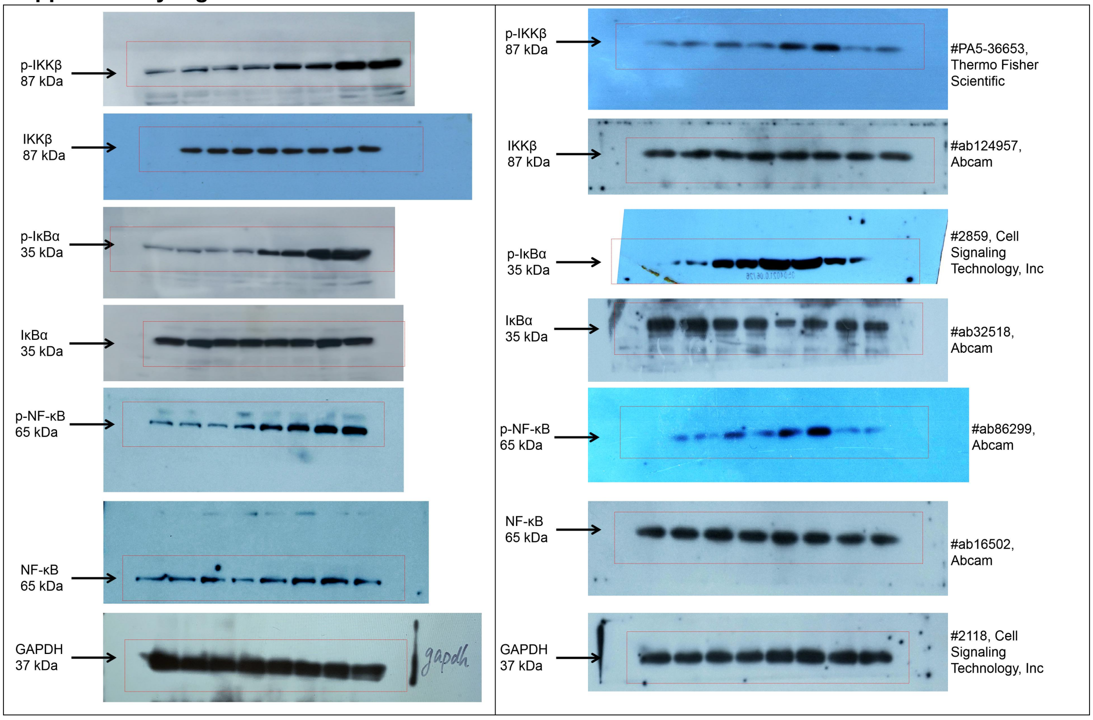

Supplementary Figure 7d

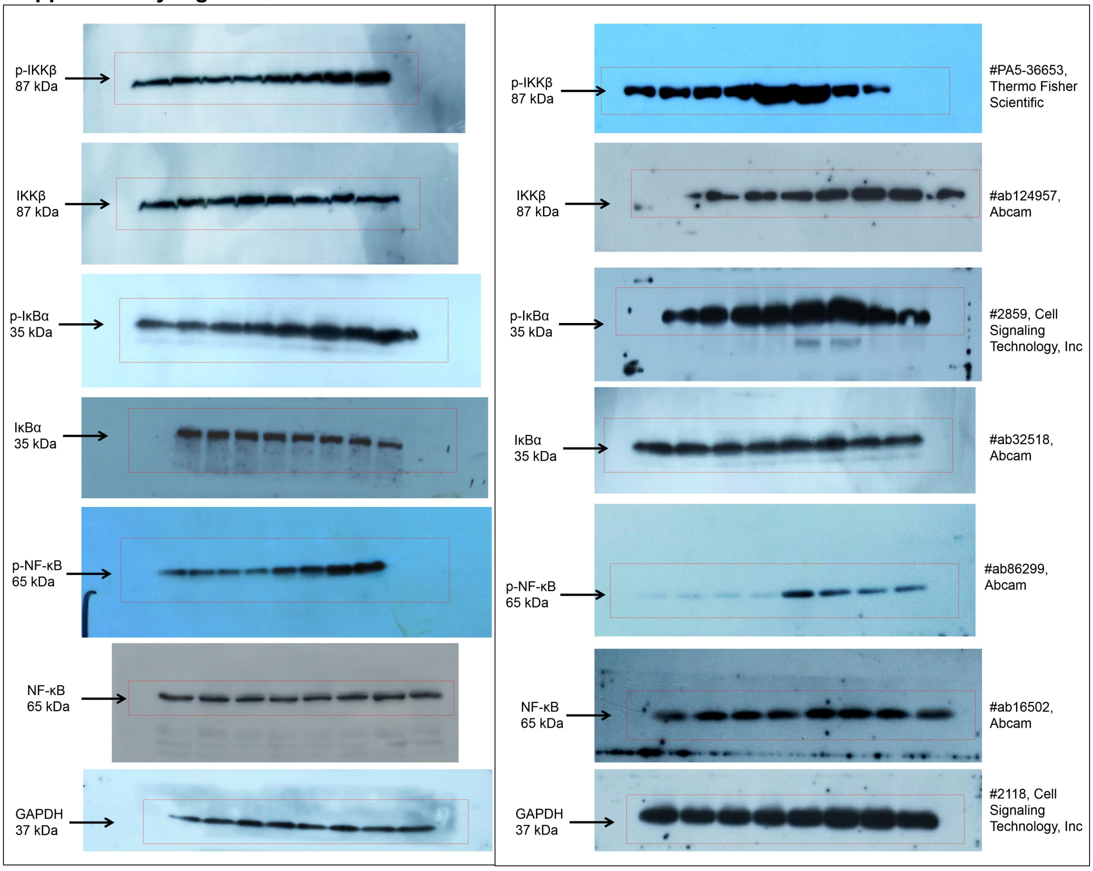

Supplementary Figure 8a

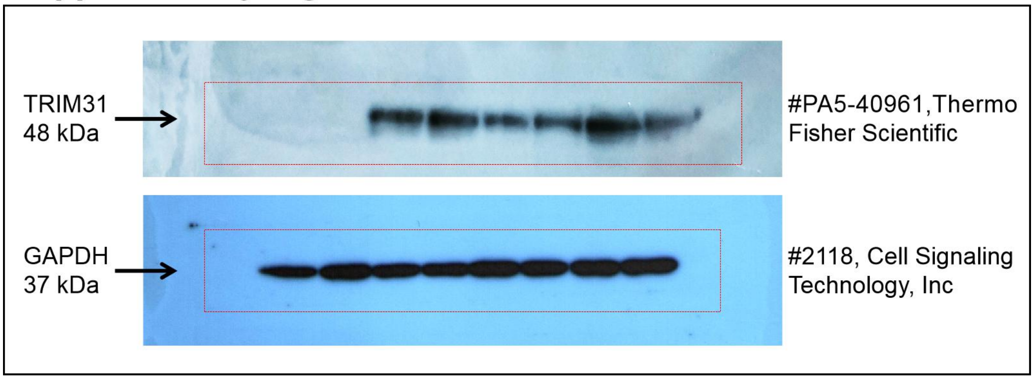

Supplementary Figure 9a

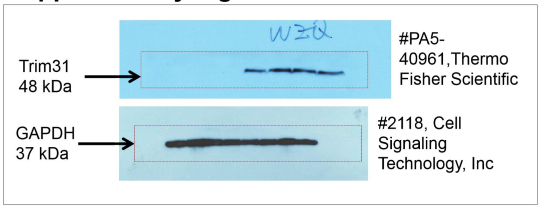

Supplementary Figure 9j

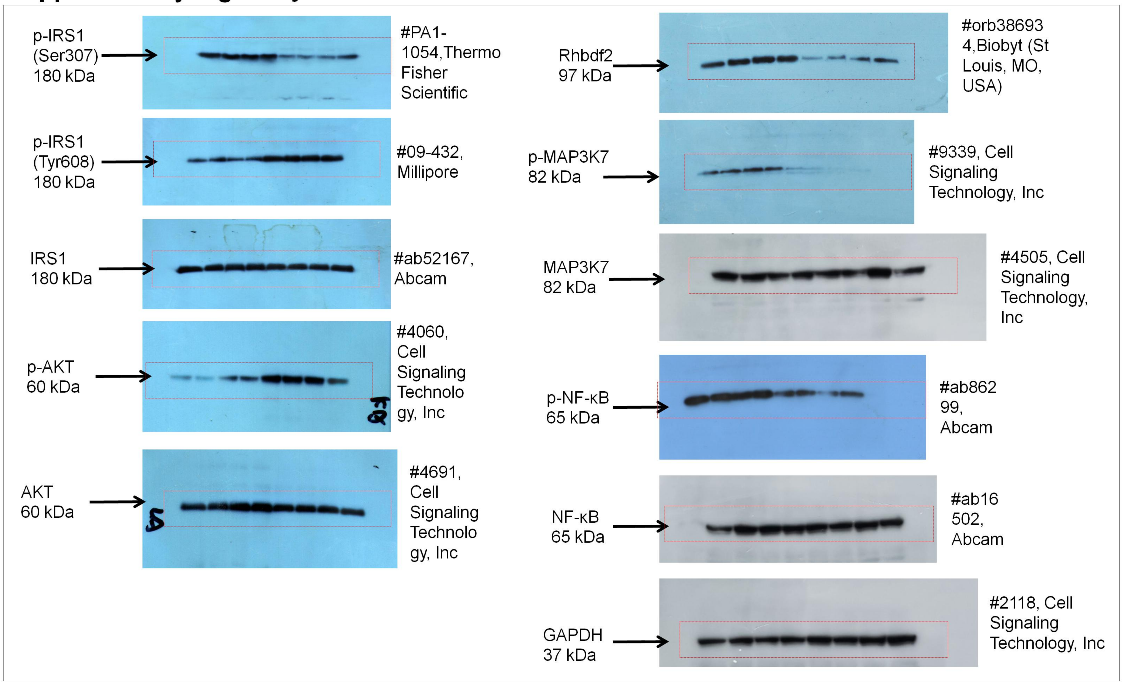

Supplementary Figure 10a

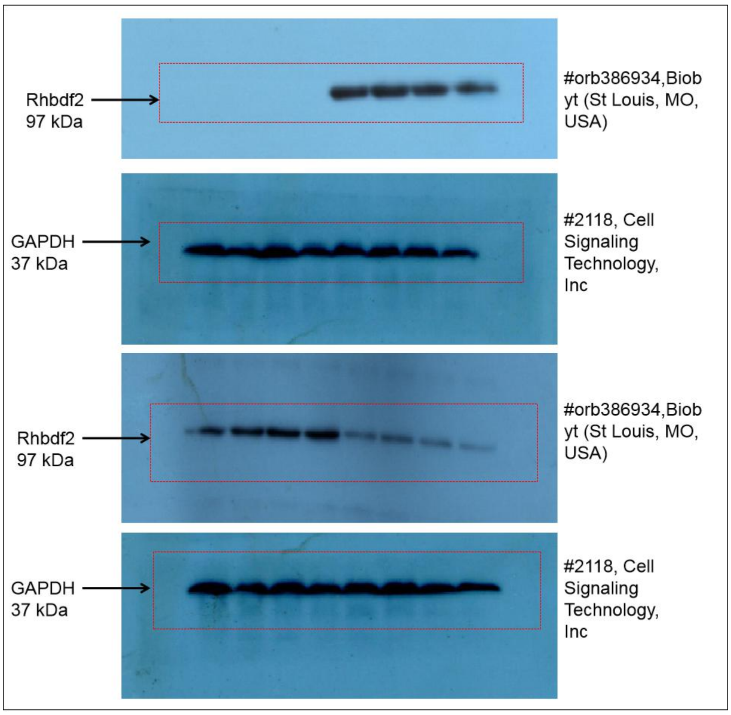

Supplementary Figure 11i

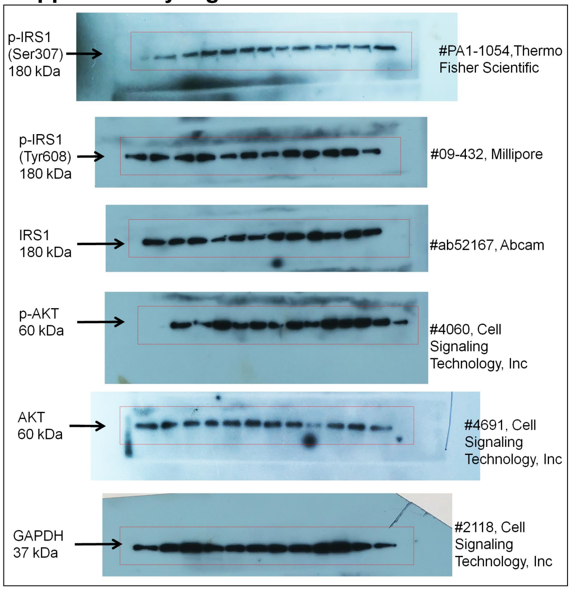

Supplementary Figure 11j

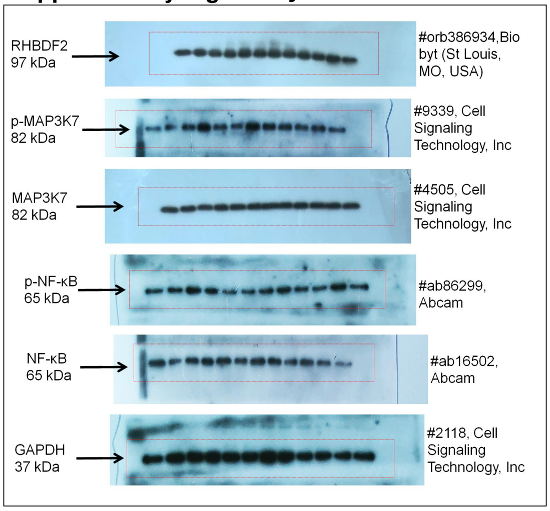

Supplementary Figure 12a

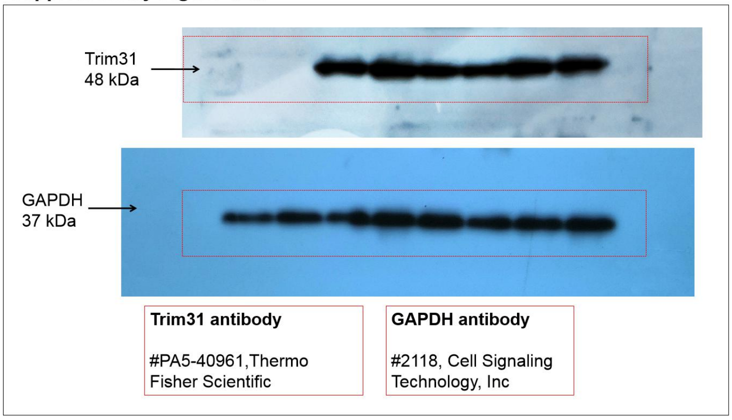

Supplementary Figure 13a

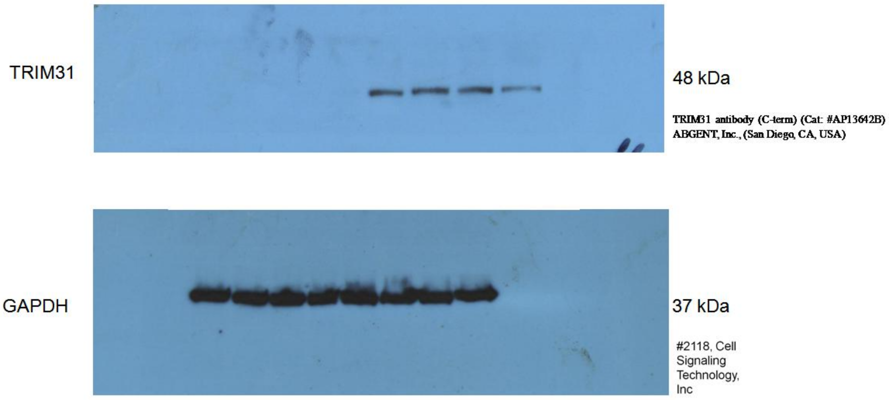

Supplementary Figure 14a

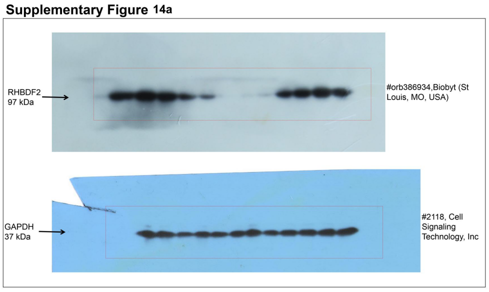

Supplementary Figure 14g

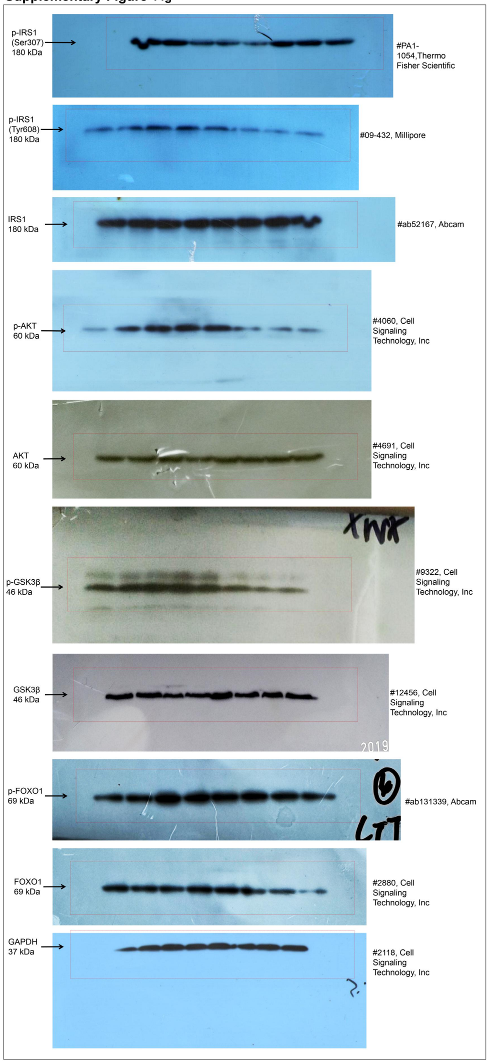

Supplementary Figure 14h

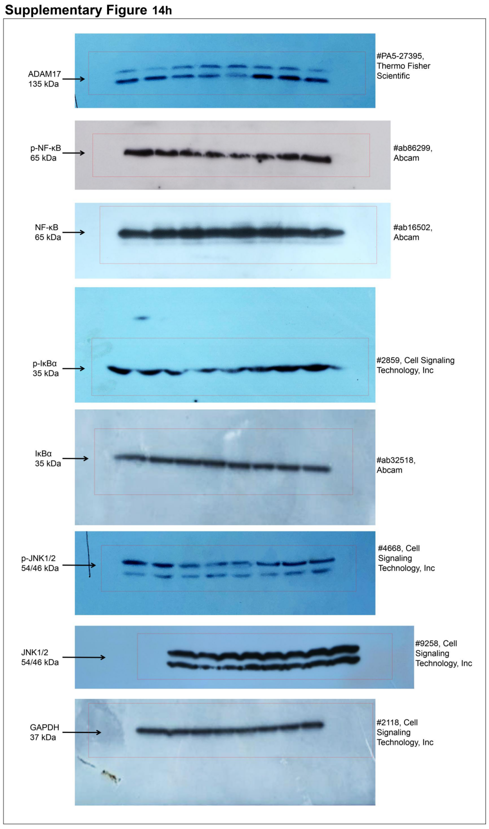

Supplementary Figure 15e

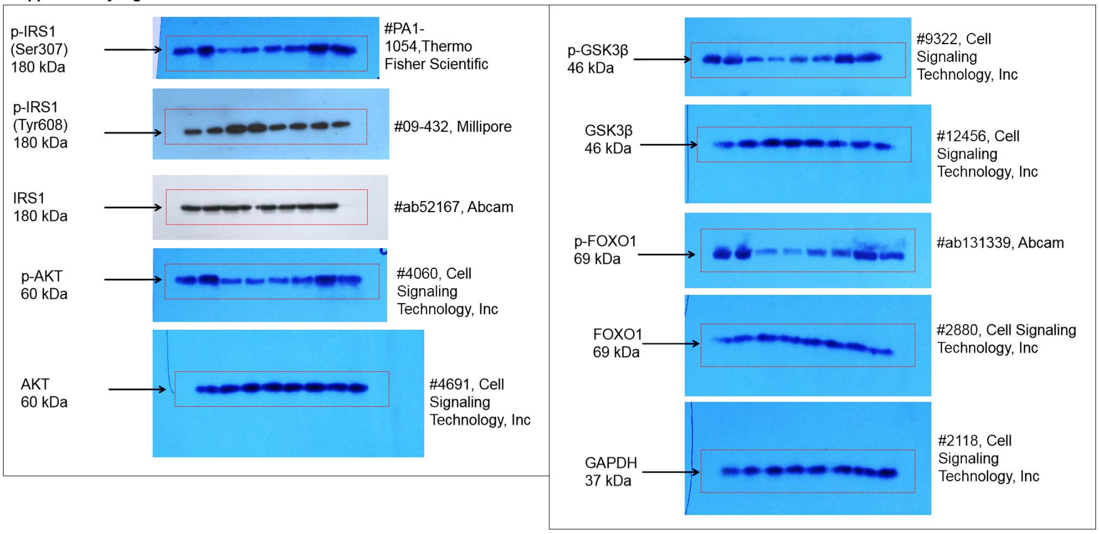

Supplementary Figure 16

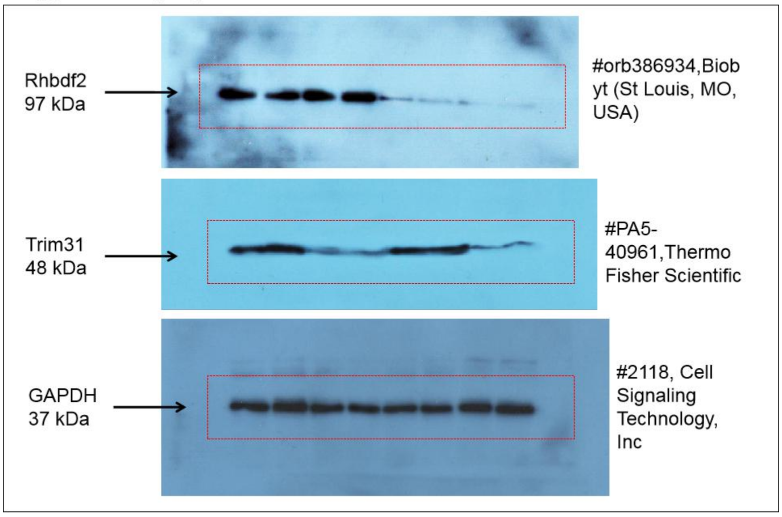

Supplementary Figure 17b

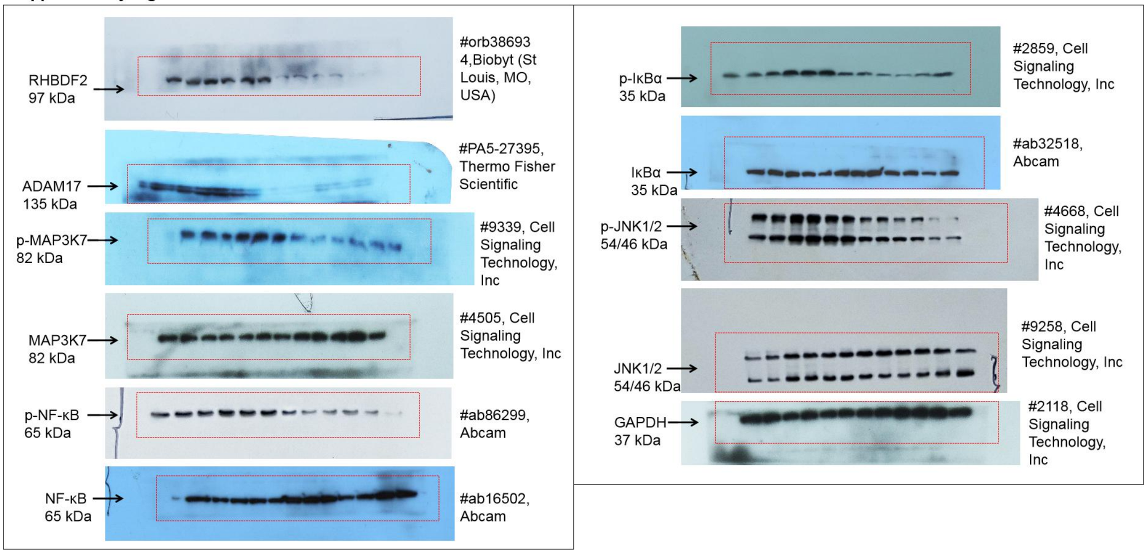

Supplement: Supplementary file 1 — Supplementary Information [file 41467_2022_28641_MOESM1_ESM.pdf]
