# Supplementary material for: The E3 ubiquitin-protein ligase Trim31 alleviates non-alcoholic fatty liver disease by targeting Rhbdf2 in mouse hepatocytes
Source: Nat Commun. 2022 Feb 25;13:1052. doi: 10.1038/s41467-022-28641-w (PMC8881609; doi:10.1038/s41467-022-28641-w)
Supplement: Supplementary file 3 — Reporting Summary [file 41467_2022_28641_MOESM3_ESM.pdf]

nature portfolio

Corresponding author(s): NCOMMS-21-07959B

Last updated by author(s): Dec 11, 2021

Reporting Summary

Nature Portfolio wishes to improve the reproducibility of the work that we publish. This form provides structure for consistency and transparency in reporting. For further information on Nature Portfolio policies, see our Editorial Policies and the Editorial Policy Checklist.

Statistics

For all statistical analyses, confirm that the following items are present in the figure legend, table legend, main text, or Methods section.

n/a	Confirmed	
☐	☒	The exact sample size (*n*) for each experimental group/condition, given as a discrete number and unit of measurement
☐	☒	A statement on whether measurements were taken from distinct samples or whether the same sample was measured repeatedly
☐	☒	The statistical test(s) used AND whether they are one- or two-sided<br>*Only common tests should be described solely by name; describe more complex techniques in the Methods section.*
☐	☒	A description of all covariates tested
☐	☒	A description of any assumptions or corrections, such as tests of normality and adjustment for multiple comparisons
☐	☒	A full description of the statistical parameters including central tendency (e.g. means) or other basic estimates (e.g. regression coefficient) AND variation (e.g. standard deviation) or associated estimates of uncertainty (e.g. confidence intervals)
☐	☒	For null hypothesis testing, the test statistic (e.g. *F*, *t*, *r*) with confidence intervals, effect sizes, degrees of freedom and *P* value noted<br>*Give P values as exact values whenever suitable.*
☒	☐	For Bayesian analysis, information on the choice of priors and Markov chain Monte Carlo settings
☒	☐	For hierarchical and complex designs, identification of the appropriate level for tests and full reporting of outcomes
☒	☐	Estimates of effect sizes (e.g. Cohen's *d*, Pearson's *r*), indicating how they were calculated

Our web collection on statistics for biologists contains articles on many of the points above.

Software and code

Policy information about availability of computer code

Data collection	Microsoft Excel & Word-Microsoft Office 2017; Image J 1.52g, NIH; EndNote X9, Thomson Router; R (V 2.15.2); Olympus Confocal FV3000, Olympus; Olympus fluorescence microscope IX70, Olympus; Olympus optical microscope CX23, Olympus; ACCU-CHEK Performa, Roche Diabetes Care GmbH; ABI PRISM 7900HT Detection Systems, Applied Biosystems
Data analysis	SPSS (Statistical Product and Service Solutions) Statistics Software (Version 26.0.0.2, Mac OS X Snow Leopard; IBM);<br>Image (Version J 1.52g, NIH) was used for quantification of positive-staining cells in the immunofluorescence staining and cell diameter of adipocytes;<br>ABI PRISM 7900HT detection systems was used for collection of qPCR data;<br>GraphPad Prism Software (Version 8.2.0, Mac OS X Snow Leopard; Graph Pad Software) was used for data analysis.

For manuscripts utilizing custom algorithms or software that are central to the research but not yet described in published literature, software must be made available to editors and reviewers. We strongly encourage code deposition in a community repository (e.g. GitHub). See the Nature Portfolio guidelines for submitting code & software for further information.

Data

Policy information about availability of data

All manuscripts must include a data availability statement. This statement should provide the following information, where applicable:

- Accession codes, unique identifiers, or web links for publicly available datasets
- A description of any restrictions on data availability
- For clinical datasets or third party data, please ensure that the statement adheres to our policy

All the raw data used in the current work are available upon request.

Field-specific reporting

Please select the one below that is the best fit for your research. If you are not sure, read the appropriate sections before making your selection.

- [x] Life sciences
- [ ] Behavioural & social sciences
- [ ] Ecological, evolutionary & environmental sciences

For a reference copy of the document with all sections, see nature.com/documents/nr-reporting-summary-flat.pdf

Life sciences study design

All studies must disclose on these points even when the disclosure is negative.

Sample size	Sample size was chosen taking in consideration the means of the target values between the experimental group and the control group, the mean standard error and the statistical analysis used. For animal studies, sample size was defined on the basis of past experience with the models, to allow a power ≥80% at the 5% significance level. For ethical reasons, the minimum number of animals necessary to achieve the scientific objectives was used.
Data exclusions	No data were excluded in the current work when performing the final statistical analysis,which have been provided state in "Statistical analysis" section.
Replication	All in vitro experiments were performed in triplicate unless specified in the figure legends. The detailed replication of each experiments has been provided in Figure Legend.
Randomization	Animals were allocated randomly to each treatment group. Different treatment groups were processed identically, and animals in different treatment groups were exposed to the same environment.
Blinding	The investigators were unaware of the experimental groups in all the quantifications.

Reporting for specific materials, systems and methods

We require information from authors about some types of materials, experimental systems and methods used in many studies. Here, indicate whether each material, system or method listed is relevant to your study. If you are not sure if a list item applies to your research, read the appropriate section before selecting a response.

Materials & experimental systems

n/a	Involved in the study	
[ ]	[x]	Antibodies
[ ]	[x]	Eukaryotic cell lines
[x]	[ ]	Palaeontology and archaeology
[ ]	[x]	Animals and other organisms
[ ]	[x]	Human research participants
[x]	[ ]	Clinical data
[x]	[ ]	Dual use research of concern

Methods

n/a	Involved in the study	
[x]	[ ]	ChIP-seq
[x]	[ ]	Flow cytometry
[x]	[ ]	MRI-based neuroimaging

Antibodies

Antibodies used	The primary antibodies used in the work and against the following proteins were obtained from Cell Signaling Technology, Inc (CST): anti-GAPDH (#2118, dilution 1:10000), p-AKT (#4060, dilution 1:1000), AKT (#4691, dilution 1:1000), p-GSK3β (#9322, dilution 1:1000), GSK3β (#12456, dilution 1:1000), FOXO1 (#2880, dilution 1:1000), anti-p-JNK (#4668, dilution 1:1000), anti-JNK (#9258, dilution 1:1000), anti-MAP3K7 (#4505, dilution 1:1000), anti-p-MAP3K7 (#9339, dilution 1:1000) and p-IκBα (#2859, dilution 1:1000). Antibodies against anti-TNFR1 (#ab223352, dilution 1:1000), anti-TNFR2 (#ab109322, dilution 1:1000), anti-MKK7 (#ab52618, dilution 1:1000), anti-p-MKK7 (#ab192592, dilution 1:1000), anti-c-Jun (#ab40766, dilution 1:1000), anti-p-c-Jun (#ab32385, dilution 1:1000), anti-IRS-1 (#ab52167, dilution 1:1000), anti-NF-κB (#ab16502, dilution 1:1000), anti-p-NF-κB (#ab86299, dilution 1:1000), p-FOXO1 (#ab131339, dilution 1:1000), anti-IκBα (#ab32518, dilution 1:1000), anti-IKKβ (#ab124957, dilution 1:1000) and anti-Rhbdf2 (#ab116139, dilution 1:1000) were purchased from Abcam (Cambridge, MA, USA). In addition, the antibodies against anti-p-MAP3K7 (#PA5-99340, dilution 1:1000), anti-p-IKKβ (#PA5-36653, dilution 1:1000), anti-Trim31 (#PA5-40961, dilution 1:1000), anti-ADAM17 (#PA5-27395, dilution 1:1000) and anti-p-IRS-1(Ser307) (#PA1-1054, dilution 1:1000) were obtained from Thermo Fisher Scientific (Waltham, MA, USA). The antibody against anti-p-IRS-1(Tyr608) (#09-432, dilution 1:1000) was purchased from Millipore. The antibody against anti-PEPCK (#sc-271029, dilution 1:1000) was purchased from Santa Cruz Biotechnology. The antibodies including anti-Rhbdf2 (#orb386934, dilution 1:1000) and anti-G6Pase (#ARP44223-P050, dilution 1:1000) were obtained from Biobyt (St Louis, MO, USA) and Aviva Systems Biology Corporation (San Diego, CA, USA), respectively. Moreover, antibodies against anti-HA (Abcam, #ab9110, dilution 1:1000), anti-Flag (Thermo Fisher Scientific, #MA1-91878, dilution 1:1000), anti-Myc (Abcam, #ab9106, dilution 1:1000), Anti-Ub (Abcam, #ab134953, dilution 1:1000), anti-Ub (linkage-specific K48) (Abcam, #ab140601, dilution 1:1000) and anti-Ub (linkage-specific K63) (Abcam, #ab179434, dilution 1:1000) were also used in the current study. The HRP-conjugated secondary antibodies (Abcam) with 1:10,000 dilution was used in immunoblotting assay for visualization.

Validation	All antibodies used in our study have been validated and detailed information could be found on the websites from manufactures as listed below:<br>anti-GAPDH (#2118, dilution 1:10000):https://www.cellsignal.com/products/primary-antibodies/gapdh-14c10-rabbit-mab/2118?site-search-type=Products&N=4294956287&Ntt=%232118&fromPage=plp&_requestid=4039564<br>p-AKT (#4060, dilution 1:1000):https://www.cellsignal.com/products/primary-antibodies/phospho-akt-ser473-d9e-xp-rabbit-mab/4060?site-search-type=Products&N=4294956287&Ntt=%234060&fromPage=plp&_requestid=4039751<br>AKT (#4691, dilution 1:1000): https://www.cellsignal.cn/products/primary-antibodies/akt-pan-c67e7-rabbit-mab/4691?site-search-type=Products&N=4294956287&Ntt=%234691&fromPage=plp&_requestid=4039864<br>p-GSK3β (#9322, dilution 1:1000): https://www.cellsignal.cn/products/primary-antibodies/phospho-gsk-3-beta-ser9-d3a4-rabbit-mab/9322?site-search-type=Products&N=4294956287&Ntt=%239322&fromPage=plp&_requestid=4127339<br>GSK3β (#12456, dilution 1:1000): https://www.cellsignal.cn/products/primary-antibodies/gsk-3b-d5c5z-xp-rabbit-mab/12456?site-search-type=Products&N=4294956287&Ntt=%2312456&fromPage=plp&_requestid=4127461<br>FOXO1 (#2880, dilution 1:1000): https://www.cellsignal.cn/products/primary-antibodies/foxo1-c29h4-rabbit-mab/2880?site-search-type=Products&N=4294956287&Ntt=%232880&fromPage=plp&_requestid=4127561<br>anti-p-JNK (#4668, dilution 1:1000): https://www.cellsignal.cn/products/primary-antibodies/phospho-sapk-jnk-thr183-tyr185-81e11-rabbit-mab/4668?site-search-type=Products&N=4294956287&Ntt=%234668&fromPage=plp&_requestid=4127595<br>anti-JNK (#9258, dilution 1:1000): https://www.cellsignal.cn/products/primary-antibodies/jnk2-56g8-rabbit-mab/9258?site-search-type=Products&N=4294956287&Ntt=%239258&fromPage=plp&_requestid=4127728<br>anti-MAP3K7 (#4505, dilution 1:1000) ： https://www.cellsignal.cn/products/primary-antibodies/tak1-antibody/4505?site-search-type=Products&N=4294956287&Ntt=%234505&fromPage=plp&_requestid=4127794<br>anti-p-MAP3K7 (#9339, dilution 1:1000) ： https://www.cellsignal.cn/products/primary-antibodies/phospho-tak1-ser412-antibody/9339?site-search-type=Products&N=4294956287&Ntt=%239339&fromPage=plp&_requestid=4127830<br>p-IκBα (#2859, dilution 1:1000) ： https://www.cellsignal.cn/products/primary-antibodies/phospho-ikba-ser32-14d4-rabbit-mab/2859?site-search-type=Products&N=4294956287&Ntt=%232859&fromPage=plp&_requestid=4127859<br>All the antibodies used are produced by trusted producers and validation protocols with WB, IP and IHC/IF is available in the website of the companies as well as in previously published papers.

Eukaryotic cell lines

Policy information about cell lines

Cell line source(s)	Human L02 cells were purchased from the Type Culture Collection of the Chinese Academy of Sciences (Shanghai, China), which have been provided in "Cell Culture and Treatment" section.
Authentication	Human L02 cell line were verified by short tandem-repeat DNA profiling before the study.
Mycoplasma contamination	Human L02 cell line were used immediately after being received he Type Culture Collection of the Chinese Academy of Sciences (Shanghai, China) and certified as Mycoplasma free.
Commonly misidentified lines (See ICLAC register)	No misidentified cell lines were used in this study.

Animals and other organisms

Policy information about studies involving animals; ARRIVE guidelines recommended for reporting animal research

Laboratory animals	Trim31flox/flox mice based on C57BL/6N background were generated using CRISPR/Cas9-mediated genome engineering system. Exons 4 and 5 of Trim31 were then selected as conditional knockout region (CKO). In brief, the chosen exons of Trim31 were flanked by loxP sites, and therefore two single guide RNAs (gRNA1 and gRNA2) targeting Trim31 introns were designed. The targeting vector containing Trim31 exon 4 and 5 flanked by two loxP sites and the two homology arms were used as the template. The targeting vector, guide RNA1 and guide RNA2 and Cas9 mRNAs were co-injected into fertilized eggs for CKO mouse production. The obtained mice, which had exon 4 and 5 flanked by two loxP sites on one allele, were used to construct Trim31flox/flox mice. Hepatocyte-specific Trim31 deletion (THKO) mice were created by mating Trim31flox/flox mice with albumin-Cre (Alb-Cre) mice (Jackson Laboratory, Bar Harbor, Maine, USA). A simple schematic diagram has been indicated in Supplementary Fig. S2a. Trim31flox/flox mice littermates were used in the study as controls for the obtained THKO mice.<br>The hepatocyte-specific Rhbdf2-knockout (RHKO) mice were also created using CRISPR/Cas9 system by specifically ablating the 4th exon of Rhbdf2 in hepatocytes. Detailed protocols and information regarding the establishment and genotype determination of these mice have been described previously 34. In addition, the hepatocyte-specific Trim31 and Rhbdf2 double deletion (DHKO) mice were generated by crossing Trim31flox/flox mice with RHKO mice.<br>Conditional Trim31 transgenic (TG) mice were established by micro-injecting CAG-loxP-CAT-loxP-Trim31 into fertilized eggs isolated from C57BL/6 mice. The obtained pups were then genotyped by PCR followed by sequencing analysis. The obtained mice were identified by PCR analysis of tail genomic DNA. The offspring of these TG mice were mated with the Alb-Cre mice to establish hepatocyte-specific Trim31 transgenic (THTG) mice. The corresponding littermates without Trim31 overexpression in hepatocytes were used as controls (NTG). Additionally, all the other normal wild-type (WT) C57BL/6N mice used in the current study were purchased from Beijing Vital River Laboratory Animal Technology Co., Ltd. (Beijing, China).
Wild animals	All animal procedures and protocols were approved by the Animal Care and Use Committee of all participating Units. Prior to all experiments proper starts, the mice were subjected to adapt to the living environment for 7 days. The mice were housed in a constant temperature, humidity (controlled by GREE central air-conditioner, #GMV-Pd250W/NaB-N1, China) and pathogen-free-controlled environment (25°C ± 2°C, 50%-60%) cage with a standard 12 h light/12 h dark cycle, plenty of water and food (pathogen-free) in their cages. The 6–8-week-old WT male mice (total 15) were fed with high fat diet (HFD) fodder (20% kcal protein, 60 kcal% fat and 20% kcal carbohydrate, #D12492; Research Diets, New Brunswick, NJ, USA) for 16 weeks to induce fatty liver. The additional

	WT mice (total 15) were fed with a standard normal chow diet (20% kcal protein, 10 kcal% fat and 70% kcal carbohydrate, #D12450H; Research Diets, New Brunswick, NJ, USA) for 16 weeks to be served as control group (NCD).
Field-collected samples	No field-collected samples were used.
Ethics oversight	All mouse experiments and procedures were reviewed and approved by the Institutional Animal Care and Use Committee (IACUC) at the Chongqing Key Laboratory of Medicinal Resources in the Three Gorges Reservoir Region and other participating units.

Note that full information on the approval of the study protocol must also be provided in the manuscript.

Human research participants

Policy information about studies involving human research participants

Population characteristics	Human liver tissue samples were collected painlessly from adult patients with nonalcoholic fatty liver disease who underwent liver transplantation or liver biopsy. The corresponding control liver tissue was harvested from the donor who could not be used for liver transplantation due to non-hepatic reasons. Written informed consent was integrally obtained from the liver donors and their families. The patient characteristics and liver injury-associated serology were showed in Supplementary table 1.
Recruitment	The descriptions of characteristics of human samples have been provided in the "Human Liver Tissue Samples" section.
Ethics oversight	All procedures associating with human subjects used in this study were based on Declaration of Helsinki, and completely permitted by the Academic Research Ethics Committee in Chongqing Key Laboratory of Medicinal Resources in the Three Gorges Reservoir Region and other participating units.

Note that full information on the approval of the study protocol must also be provided in the manuscript.
